# The cannabinoid-1 receptor is abundantly expressed in striatal striosomes and striosome-dendron bouquets of the substantia nigra

**DOI:** 10.1371/journal.pone.0191436

**Published:** 2018-02-21

**Authors:** Margaret I. Davis, Jill R. Crittenden, Austin Y. Feng, David A. Kupferschmidt, Alipi Naydenov, Nephi Stella, Ann M. Graybiel, David M. Lovinger

**Affiliations:** 1 Laboratory for Integrative Neuroscience, National Institute on Alcohol Abuse and Alcoholism, National Institutes of Health, Bethesda, Maryland, United States of America; 2 McGovern Institute for Brain Research, Department of Brain and Cognitive Sciences, Massachusetts Institute of Technology, Cambridge, Massachusetts, United States of America; 3 Laboratory of Brain Immunology, University of Washington School of Medicine, Seattle, Washington, United States of America; Hudson Institute, AUSTRALIA

## Abstract

Presynaptic cannabinoid-1 receptors (CB_1_-R) bind endogenous and exogenous cannabinoids to modulate neurotransmitter release. CB_1_-Rs are expressed throughout the basal ganglia, including striatum and substantia nigra, where they play a role in learning and control of motivated actions. However, the pattern of CB_1_-R expression across different striatal compartments, microcircuits and efferent targets, and the contribution of different CB_1_-R-expressing neurons to this pattern, are unclear. We use a combination of conventional techniques and novel genetic models to evaluate CB_1_-R expression in striosome (patch) and matrix compartments of the striatum, and in nigral targets of striatal medium spiny projection neurons (MSNs). CB_1_-R protein and mRNA follow a descending dorsolateral-to-ventromedial intensity gradient in the caudal striatum, with elevated expression in striosomes relative to the surrounding matrix. The lateral predominance of striosome CB_1_-Rs contrasts with that of the classical striosomal marker, the mu opioid receptor (MOR), which is expressed most prominently in rostromedial striosomes. The dorsolateral-to-ventromedial CB_1_-R gradient is similar to Drd2 dopamine receptor immunoreactivity and opposite to Substance P. This topology of CB_1_-R expression is maintained downstream in the globus pallidus and substantia nigra. Dense CB_1_-R-expressing striatonigral fibers extend dorsally within the substantia nigra pars reticulata, and colocalize with bundles of ventrally extending, striosome-targeted, dendrites of dopamine-containing neurons in the substantia nigra pars compacta (striosome-dendron bouquets). Within striatum, CB_1_-Rs colocalize with fluorescently labeled MSN collaterals within the striosomes. Cre recombinase-mediated deletion of CB_1_-Rs from cortical projection neurons or MSNs, and MSN-selective reintroduction of CB_1_-Rs in knockout mice, demonstrate that the principal source of CB_1_-Rs in dorsolateral striosomes is local MSN collaterals. These data suggest a role for CB_1_-Rs in caudal dorsolateral striosome collaterals and striosome-dendron bouquet projections to lateral substantia nigra, where they are anatomically poised to mediate presynaptic disinhibition of both striosomal MSNs and midbrain dopamine neurons in response to endocannabinoids and cannabinomimetics.

## Introduction

Endocannabinoids and their receptors participate in a variety of pathways and behaviors, including pain perception, appetite, learning, movement control, habit formation, and addiction [1, 2]. Generally released from postsynaptic neuronal elements in the brain, endocannabinoids signal retrogradely to suppress synaptic transmission through the presynaptic G-protein coupled receptor (GPCR) cannabinoid-1 receptor (CB_1_-R) [1, 2]. Although several endocannabinoid-activated GPCRs and ion channels have been identified in the nervous system, the two most thoroughly characterized cannabinoid receptors are CB_1_-R and CB_2_-R [2–4]. CB_1_-Rs are the major pharmacological target mediating the psychoactive properties of delta-9-tetrahydrocannabinol and many abused synthetic cannabinomimetics, while CB_2_-Rs are less widely expressed in the central nervous system. Many novel receptors, and endocannabinoids with differing receptor affinities, are also present in the central nervous system [3].

Within the basal ganglia circuitry, CB_1_-Rs are implicated in modulation of synaptic transmission and plasticity as well as indirect regulation of dopamine release [1, 5, 6]. CB_1_-Rs are expressed in a dorsolateral-to-ventromedial gradient for both protein and mRNA [7–14]. Evidence from receptor binding, in situ hybridization, and immunostaining have hinted that CB_1-_Rs may also be enriched in striosome compartments within the striatum [2, 7, 9, 10]. The enrichment of CB_1_-Rs in lateral striatal sector is consistent with differential roles for CB_1_-Rs in behaviors preferentially controlled by medial vs. lateral striatal sectors, and with data implicating CB_1_-Rs in the transition from goal-directed to habitual behaviors that occurs with behavioral repetition [15–20]; however, the role that CB_1_-Rs in striosomes and striosomal afferent projections may play in controlling these behaviors is unknown.

The majority of CB_1_-Rs detected in the cortex, hippocampus, and amygdala are localized to GABAergic axons [4, 14, 21]; this is also true of striatum where Cre-mediated reintroduction into a CB_1_-R null background indicates a modest contribution from cortical glutamatergic sources [14]. Within the basal ganglia, CB_1_-Rs are found on axons on MSNs, cortical axons, and GABAergic interneurons, but they are notably absent from dopamine terminals and cholinergic interneurons [4, 6, 8–10, 22]. Expression of CB_1_-R mRNA is found in striatal Drd1- and Drd2-dopamine receptor positive MSNs [8–10, 13], and CB_1_-R protein is abundant in their respective outputs to the entopeduncular nucleus (GPi), substantia nigra pars reticulata (SNpr) and the globus pallidus (GP) [2, 4, 10]. Although heterogeneous distribution of CB_1_-Rs in relation to the striosome and matrix compartments has been suggested [2, 7, 9, 10], patchy non-striosomal clusters (matrisomes) exist in the matrix compartment. It is therefore unclear whether this heterogeneity corresponds to conventional striosome-matrix architecture.

Striosomes of the dorsal striatum are distinguished from the extra-striosomal matrix surrounding them by their patterns of gene expression, input-output connections, developmental birthdates, and neuronal activity profiles [23]. In addition to the canonical striatal outputs carried by the direct pathway, originating in Drd1-dopamine receptor-expressing medium spiny projection neurons (MSNs), and the indirect pathway, originating in Drd2-dopamine receptor-expressing MSNs, there exists a third projection pathway originating from striosomes that directly targets dopamine-containing neurons in the midbrain [24–29]. Axons from these striosomal MSNs are intimately entwined with bundles of ventrally extending dendrites that arise from clusters of dopamine-containing neurons in the substantia nigra pars compacta (SNpc) [27]. These structures, called ‘striosome-dendron bouquets’, have been highlighted by virtue of their prominence in mice with enriched reporter-gene expression in striosome MSNs, and their minimal labeling in mice with preferential reporter gene expression in matrix MSNs [27]. Together these data support the view that striosomes have a unique projection to dopamine-containing neurons comprising these bouquets. This is in accord with findings from single-cell and tract-tracing tracing experiments that identified projections from striosomes directly to the SNpc [24–29], but see [30]. The functions of this specialized striosomal projection system have not been identified.

In parallel to their differing output circuits, striosomes and matrix have distinct though overlapping inputs. Dopamine-containing neurons in the ventral tier of the SNpc, which are targeted by striosome MSNs, form part of a circuit favoring innervation of the striosomes over the surrounding matrix [27, 31–33]. Dopamine release is heterogeneously regulated in the striosomes and matrix compartments [34–36], where cocaine application to brain slices differentially regulates dopamine release in striosomes when compared to the surrounding matrix or a different striatal sector [34, 35]. Striosomes also differ from the matrix with respect to cortical innervation in a region-specific manner. The dorsolateral matrix receives dense innervation from somatosensory and motor cortices, and the dorsomedial matrix from the cingulate cortex, whereas the rostral and medial mu opioid receptor (MOR)-enriched striosomes receive innervation from restricted parts of the prefrontal and caudal orbitofrontal cortex [23, 37–40]. This distribution raises the possibility that sensorimotor and limbic circuits may be differentially influenced by the outputs from these different striatal regions.

Striosomal circuits have been directly implicated in opioid and psychomotor stimulant reward pathways based on MOR signaling and differential gene activation by drugs of abuse [41–45]. Behaviorally, the relative activation of striosomes versus matrix has been related to abnormally repetitive behaviors induced by drugs of abuse [16, 23], and to decision-making under conditions of motivational conflict [40, 46] and stress [47]. Given the different regional contexts within which the striosomes are imbedded, groups of striosomes and their projections to the SNpc should not be considered a single unit. Different striosomes may integrate information from convergent pathways or show variable plasticity depending on their location. Therefore they should not be considered a homogeneous population, although they might serve a higher-order function integrating information from many striatal regions.

We use a variety of recently characterized striosome and matrix selective mouse lines [23, 27, 34, 35, 48, 49], Cre-mediated deletion or tagging [50], and conventional immunofluorescence to identify the neuronal source of the “patchy” CB_1_-R-expressing fibers, and confirm their position with respect to the striosome-matrix labyrinth. Cre mediated deletion, reintroduction, and the use of fluorescent protein reporters indicate that, within dorsolateral and caudal striosomes, the majority of striosomal CB_1_-R immunoreactivity can be attributed to MSN axons in a gradient reminiscent of Drd2 receptor expression, and largely inverse to the striosomal MOR expression gradient. CB_1_-R colocalized with Drd2-eGFP expressing fibers more frequently than Drd1-tdTomato expressing fibers in these striosomes. In contrast, Drd1-tdTomato- and Nr4a1-eGFP- (striosome-enriched) expressing projections to dopaminergic neurons in the ventral tier of the SNpc via dendron bouquets are highly enriched in CB_1_-Rs, while matrix enriched CalDAG-GEFI-eGFP- expressing axons within the SNpr are not. Given the density of CB_1_-Rs in both the local Drd2-enriched striosome MSN collaterals, and the Drd1-expressing striosomal terminals in the substantia nigra striosome-dendron bouquets, these results are consistent with the existence of multiple subdomains within the basal ganglia circuitry where endocannabinoids can regulate basal ganglia function through inhibition of MSN GABA release. Further studies should consider this unique subcircuit anatomy, as well as the contribution of CB_1_-Rs in striatopallidal projections, when examining cannabinoid modulation of basal ganglia function.

## Methods

### Mice

Procedures were approved by the Animal Care and Use Committees at the National Institute on Alcohol Abuse and Alcoholism (NIAAA) and Massachusetts Institute of Technology (MIT). All procedures were performed in accordance with NIH guidelines for humane and ethical treatment of animals. Wild-type C57BL/6J mice were obtained from the Jackson Laboratory (Bar Harbor, ME) and used as the background strain for these experiments. RGS9^tm1(cre)Yql^ (RGS9-Cre) mice were developed and provided by Dang and colleagues [51]. The CB_1_-R knock out mice containing a loxP-flanked stop sequence between the chicken beta-actin and a cnr1 coding sequence (in the Rosa26 locus) were crossed with MSN-selective GPR88-Cre line. After cell specific recombination, the stop site is excised, and expression is controlled by the chicken beta-actin promoter. These mice were characterized by Naydenov and colleagues [52]. Emx1-Cre (B6.129S2-Emx1^tm1(cre)Krj^/J) and GAD-Cre (Gad2^tm2(cre)Zjh^/J) mice were obtained from Jackson Labs. RGS9-Cre and GAD-Cre lines were validated by breeding hemizygous Cre mice to Cre-dependent tdTomato reporter lines (B6.Cg-Gt(ROSA)26Sor^tm14(CAG-tdTomato)Hzw^/J (Ai14) or B6.Cg-Gt(ROSA)26Sor^tm27.1(CAG-COP4*H134R/tdTomato)Hze^/J (Ai27D), respectively) from Jackson Labs. ZsGreen reporter mice (B6.Cg-Gt(ROSA)26Sor^tm6(CAG-ZsGreen1)Hze^/J) were used to characterize the Emx1-Cre mice due to unresolvable, intense expression in the tdTomato reporters. Double bacterial artificial chromosome transgenic Drd1-tdTomato:Drd2-eGFP mice were characterized by Ade and colleagues [53], obtained from Jackson Labs and GENSAT, respectively, and maintained on a C57BL/6J background. Floxed CB_1_-R mice (CB1^f/f^) were generously provided by Giovanni Marsicano and Beat Lutz. Pups from RGS9-Cre crosses with ectopic global CB_1_-R deletion detected by PCR were excluded from analysis. Hemizygous Nr4a1-eGFP (Tg(Nr4a1-EGFP)GY139Gsat/Mmucd) and hemizygous CalDAG-GEFI-eGFP (Tg(Rasgrp2-EGFP)DU111Gsat/Mmucd (CalDAG-GEFI-eGFP)) mice were obtained from GENSAT [50] and were previously described [23, 27, 34, 35, 49]. Both male and female mice were examined at 45–90 days of age in all experiments except for RGS9-Cre and Emx1-Cre deletions where experiments were performed at 25–30 days of age, a period that corresponds to the developmental peak in CB1-R expression in the striatum [12]. We provide extended datasets on the characterization of the mice used in the supplemental data (S1–S6 Figs).

### In situ hybridization

CalDAG-GEFI-eGFP mice were deeply anesthetized with Euthasol (Virbac AH Inc.; pentobarbital sodium and phenytoin sodium), the brains were removed and placed on a cold surface, and from each a broad coronal slab containing the striatum was placed into a cryo-mold containing cold Optimal Cutting Temperature (OCT) Compound (Tissue-Tek, Sakura Finetek Inc.). The tissue was then covered in OCT and the cryomold was placed into a bath of methylbutane that was pre-cooled on dry ice. The cryomold was removed from the bath as soon as the OCT became opaque, and then the encased brains were stored at -80C until sectioning. Frozen sections (10 μm) were cut with a cryostat, placed onto positively charged slides (Leica Biosystems) and stored at -80C until use. Single-plex and duplex chromogenic in situ hybridization was done exactly as described in the ACDBio RNAscope 2.5 manual with probes directed to cnr1 (#420721, ACDBio) and gfp (#400281, ACDBio). Slides were scanned at high resolution with an Aperio slide scanner (Leica Biosystems) and images were processed in Adobe Photoshop CC (Adobe Systems Inc.).

### Immunofluorescence

Mice were deeply anesthetized with isoflurane or pentobarbital and transcardially perfused with PBS followed by 4% freshly depolymerized paraformaldehyde in PBS and post-fixed for 90 minutes (for Figs designated as “MIT Protocol”, see 10.17504/protocols.io.kracv2e for detailed protocol) or overnight (all others, 10.17504/protocols.io.kz3cx8n). Coronal vibratome sections (40 μm) or glycerol cryoprotected frozen sliding microtome sections (30 μm; MIT protocol) were taken at levels extending from the prefrontal cortex to the ventral mesencephalon, and every 3^rd^ to 6^th^ section, depending on the experiment, was stained and imaged. Sections were washed in PBS-T (0.2% Triton X-100), blocked for 4 hours with 5% BSA in PBST, and incubated in primary antibodies overnight to 48 hours in a cold room on an orbital shaker. Primary antibodies are listed in Table 1. Antibodies were titrated to a concentration that yielded unsaturated detection within the linear range of staining of the entire striatum at low power on a wide field fluorescence microscope. All CB_1_-R antibodies tested (Dr. Kenneth Mackie of Indiana University, Cayman, Synaptic Systems, and Frontier Biosciences) varied significantly in titer but resulted in a similar staining pattern at this resolution. The majority of experiments presented here were performed with the goat anti-CB_1_-R primary antibody, as multi-label experiments precluded the use of the rabbit antibody. Secondary antibodies made in donkey were used for multi-label experiments with the goat anti-CB_1_-R antibody. All secondary antibodies (AF350, AF488, AF568, and AF647) were from Thermo-Fisher, except for the donkey anti-chicken antibodies, which were from Jackson Immunolabs. All secondary antibodies were titrated and tested for non-specific immunoreactivity by omitting the primary antibodies.

**Table 1:** Antibodies and sources.

Antigen	Supplier	Dilution	Catalogue and RRID numbers
Goat Anti-CB_1_-R	Frontier Biosciences	1:250–1:500	CB1-Go-Af450, RRID: AB_2571592
Rabbit Anti-CB1	Cayman (L20)	1:2000	10006590–1, RRID:AB_409026
Rabbit Anti-CB_1_-R	K.Mackie (L15)	1:1000	
Mouse Anti-CB_1_-R	Synaptic Systems	1:500	258 011, RRID:AB_2619969
Rabbit Anti-TH	Abcam	1:4000	Ab112
Rabbit Anti-TH	Thermo	1:500	AB_297840, RRID: B112 AB_297840
Rabbit Anti Drd2	Frontier	1:500	D2R-Rb-Af960, RRID: AB_2571596
Rat Anti-SubP	Abcam	1:250	ab7340, RRID:AB_305866
Chicken Anti-GFP	Abcam	1:1000	ab13970, RRID: AB_300798
Chicken Anti-GFP	Aves	1:500	GFP120, RRID AB_10000240
Rabbit Anti-MOR	Immunostar	1:4000	24216. RRID:AB_572251
Rabbit Anti-MOR	Abcam	1:500	Ab134054
Rabbit Anti-dsRed	Clontech	1:200–1:1000	632496, RRID:AB_10013483
Mouse Anti-Calbindin	Swant	1:500	300, RID: AB_10000347

Wide field mages were collected with a Zeiss Axiocam using a Zeiss Stereo Lumar microscope, Zeiss Axiozoom microscope, or Axiovert 200 microscope (10X 0.4 NA, 20X 0.8 NA objectives) as indicated in the figure legends. Epifluorescence filter sets were standard for DAPI (350 excitation, 400 long pass beam splitter, 460/50 emission), eGFP/AF488 (470/40 excitation, 495 beam splitter, 525/50 emission) and AF568/Texas red (560/40 ex, 585lp BS, 630/75 em). Confocal images were collected with an LSM510, LSM880, or an Olympus FV 1200 laser-scanning microscope as indicated in the figure legends. For Zeiss microscopes, confocal and 2-photon imaging was performed with a chameleon Ti:Sapphire (760 nm for AF350), Argon Ion (AF488), 561 nm DPSS (AF568), and HeNe (AF647) lasers. Z-series were collected with Apochromat 20x/0.8 NA, 40x/1.3 NA or Apochromat 63x/1.3 NA objectives using 0.5–1 μm steps. For images taken with the Olympus confocal, DPSS lasers (473, 559, and 635) were used for excitation. Images were collected with a 10x 0.4 NA and 60X silicon oil 1.3 NA objectives using, respectively, 2.0 and 0.5 μm and tiled to create the presented images. Maximum and average intensity projection images were generated from optical sections using Image J. Background was subtracted and contrast was adjusted for presentation.

Images were quantified using Image J and sampled regions were selected to avoid large fiber bundles, when possible. Quantification along lines was performed on raw data and intensity plots were generated using the “plot profile” macro in Image J to generate line plots. For further quantification of CB_1_-R immunoreactivity, a 100 x150 pixel box was sampled on sections between bregma 0.2 mm and -0.9 mm in the posterior striatum from six different animals (nine sections) and mean intensity quantified. Data were analyzed in Prism by two-way ANOVA using a post hoc Bonferroni’s multiple comparisons test.

## Results

### Verification of striosomal CB1-R expression in the lateral striatum

**Fig 1:**
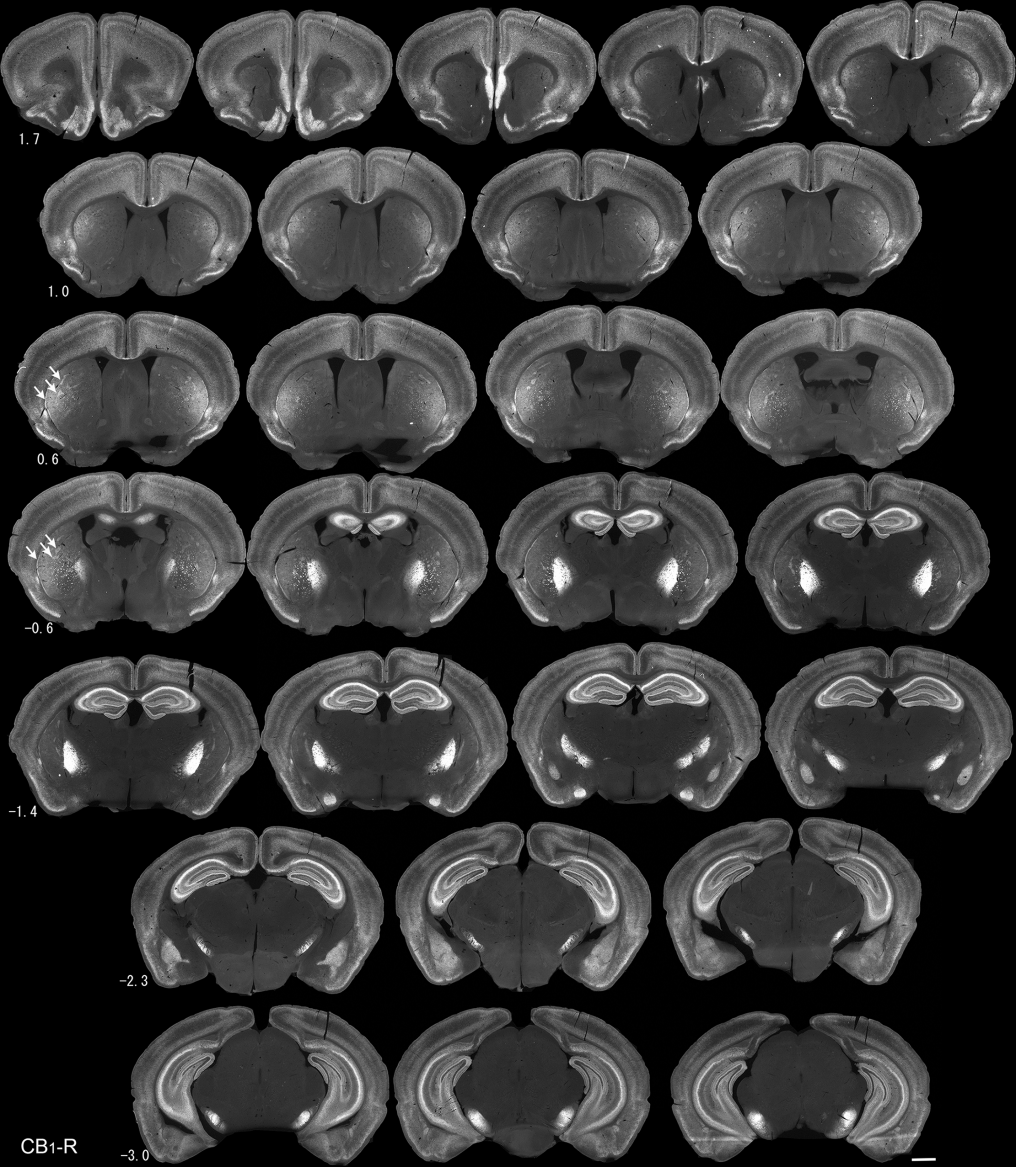
Meso-scale CB_1_-R immunoreactivity through the basal ganglia. The lateral subcallosal streak and several prominent striosomes in the lateral striatum are indicated by arrows. Numbers indicate location relative to bregma, and sections are 120 μm apart. Images were taken with the Axiozoom microscope. Scale bar is 1 mm.

In a first series of experiments, we documented the expression pattern of CB_1_-Rs in the mouse brain, focusing on the striatum, pallidum and substantia nigra using immunofluorescence. With several well-characterized antibodies against the C-terminus of the CB_1_R, we surveyed CB_1_-R distribution at 120-μm intervals, mapping coordinates relative to bregma (Fig 1, goat anti-CB_1_-R, Frontier, is shown). We observed high levels of immunoreactivity with all antibodies tested in the hippocampus, amygdala, medial prefrontal cortex, and basal ganglia, consistent with previous observations in mice and rats using immunohistochemistry, *in situ* hybridization, and receptor binding [2, 4, 7, 9–11, 21, 54]. Within the basal ganglia, a detailed meso-scale survey of CB_1_-R immunoreactivity revealed a gradient in expression that begins in the most extreme dorsolateral regions of the dorsal striatum and strengthens to encompass most of the lateral striatum, particularly at levels posterior to the horizontal limb of the anterior commissure (Fig 1). Within the striatum proper, the most intense CB_1_-R signal occurred in the subcallosal and lateral striatal streaks, diffuse patchy regions, and layers of higher CB_1_-R immunoreactivity that were most apparent in the caudal, post-commissural striatum between 0.2 mm and -0.6 mm relative to bregma. Consistent with high expression of CB_1_-Rs in MSNs, CB_1_-R immunoreactivity was particularly intense in striatal projections, including those to the lateral globus pallidus and entopeduncular nucleus (GPe and GPi) and lateral regions of the SN, retaining the topography seen in the dorsal striatum through the pathway. Only low-level diffuse CB_1_-R expression and sparse, tortuous axons were detected in the nucleus accumbens, consistent with previous observations [54].

**Fig 2:**
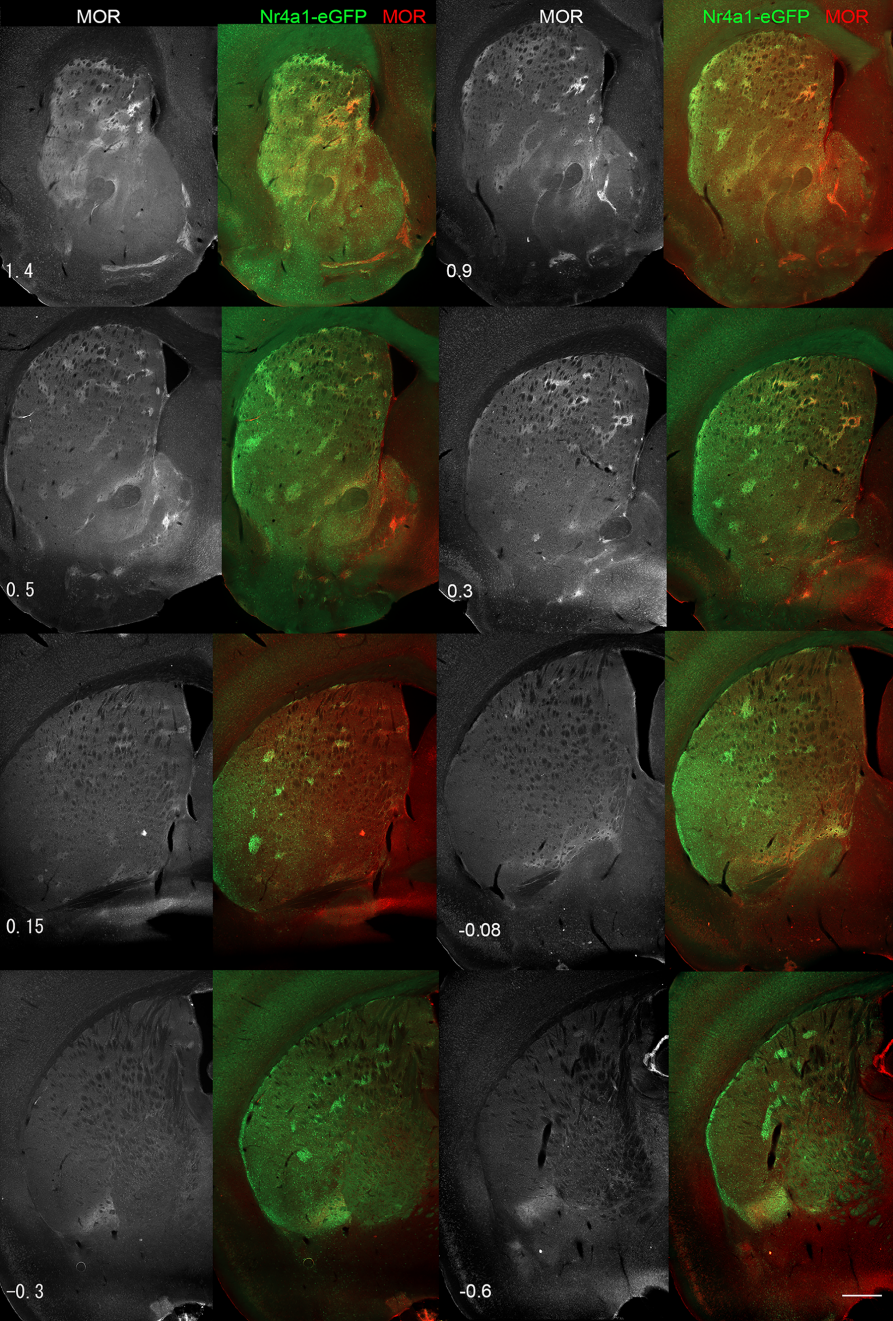
MOR expression gradient fading in caudal and lateral striosomes. Serial sections through the Nr4a1-eGFP striatum were stained for MOR at 240 μm intervals. MOR alone is shown in black and white alone or in red with Nr4a1-eGFP in the adjacent left panel. Numbers indicate the relative location relative to bregma. Images were taken with the Lumar microscope. Scale bar is 500 μm.

To confirm that these CB_1_-R-immunoreactive structures were a specific sub-group of striosomes with enhanced CB_1_-R expression, we performed double-labeling experiments at the mRNA and protein level, employing striosome- and matrix-enriched eGFP transgenic mouse lines and the prototypical striosome marker, MOR. We first identified striosomes through the extent of the striatum using the well-characterized Nr4a1-eGFP mice [34, 35, 49]. In Nr4a1-eGFP mice, in which all striosomes express elevated levels of eGFP (see S1 Fig), MOR immunoreactivity was enriched in rostral and caudal striosomes, and the degree of overlap between MOR and Nr4a1-eGFP depended on the region sampled (Fig 2). A principal difference was that Nr4a1-eGFP-positive striosomes were visible in lateral regions caudal to bregma 0.15 mm where MOR staining was weak or minimal in the lateral striatum. This is in agreement with previous observations of a gradient of MOR localization in the striatum [55].

**Fig 3:**
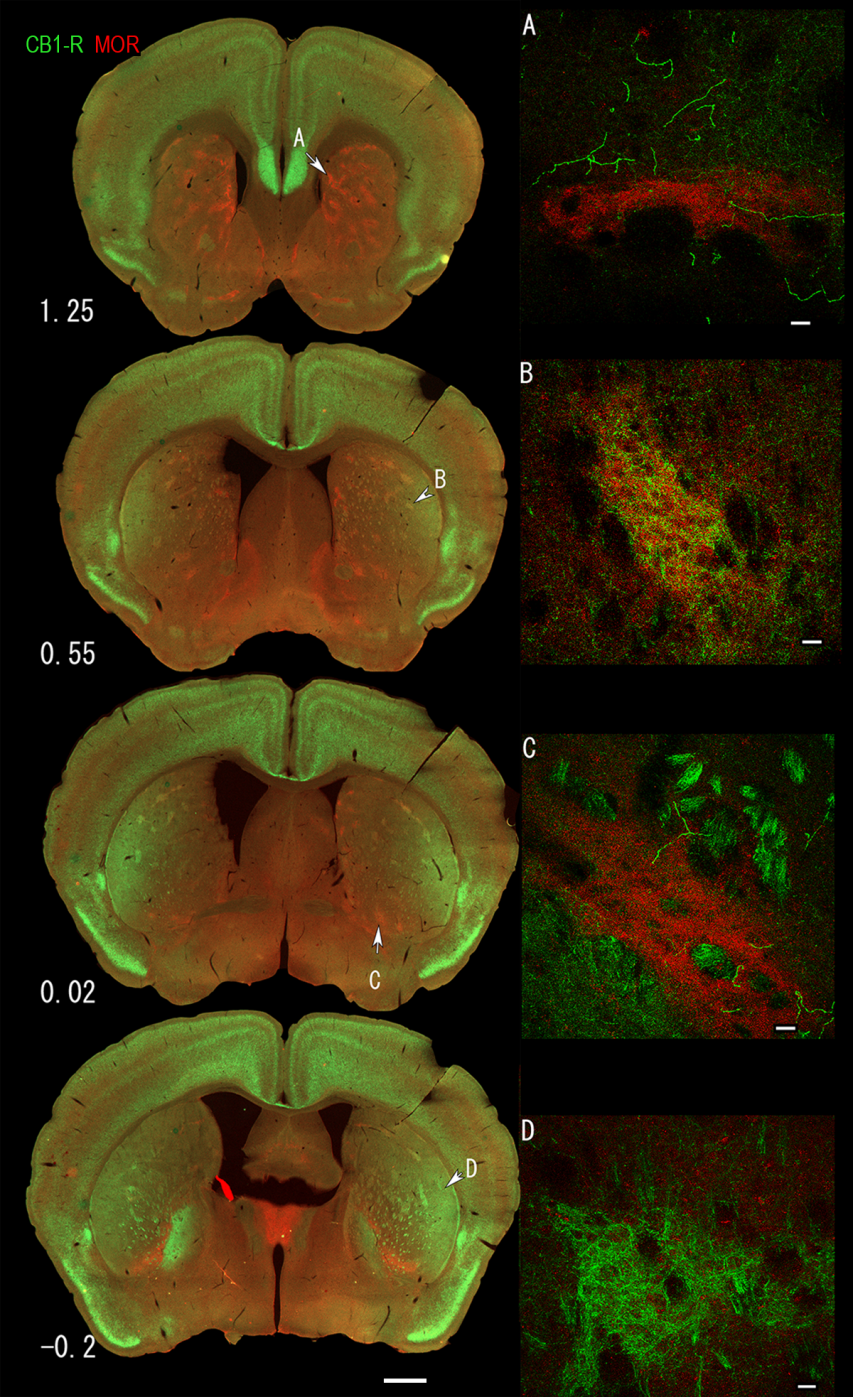
Overlap and segregation of MOR (red) and CB_1_-R (green) at different levels through the striatum. Four sections are presented between 1.25 and -0.2 mm relative to bregma (left). Examples of MOR-dominant staining in the rostral dorsomedial striatum (A), overlap between MOR and CB_1_-R in the dorsolateral striosomes (B), MOR-dominant ventral striosomes (C) and CB_1_-R-dominant striosomes (D) are presented at high power. Low power images were taken with the Axiozoom. Projection images on the right were taken with the LSM510. Scale bars are 1 mm in the left column and 20 μm in A-D.

**Fig 4:**
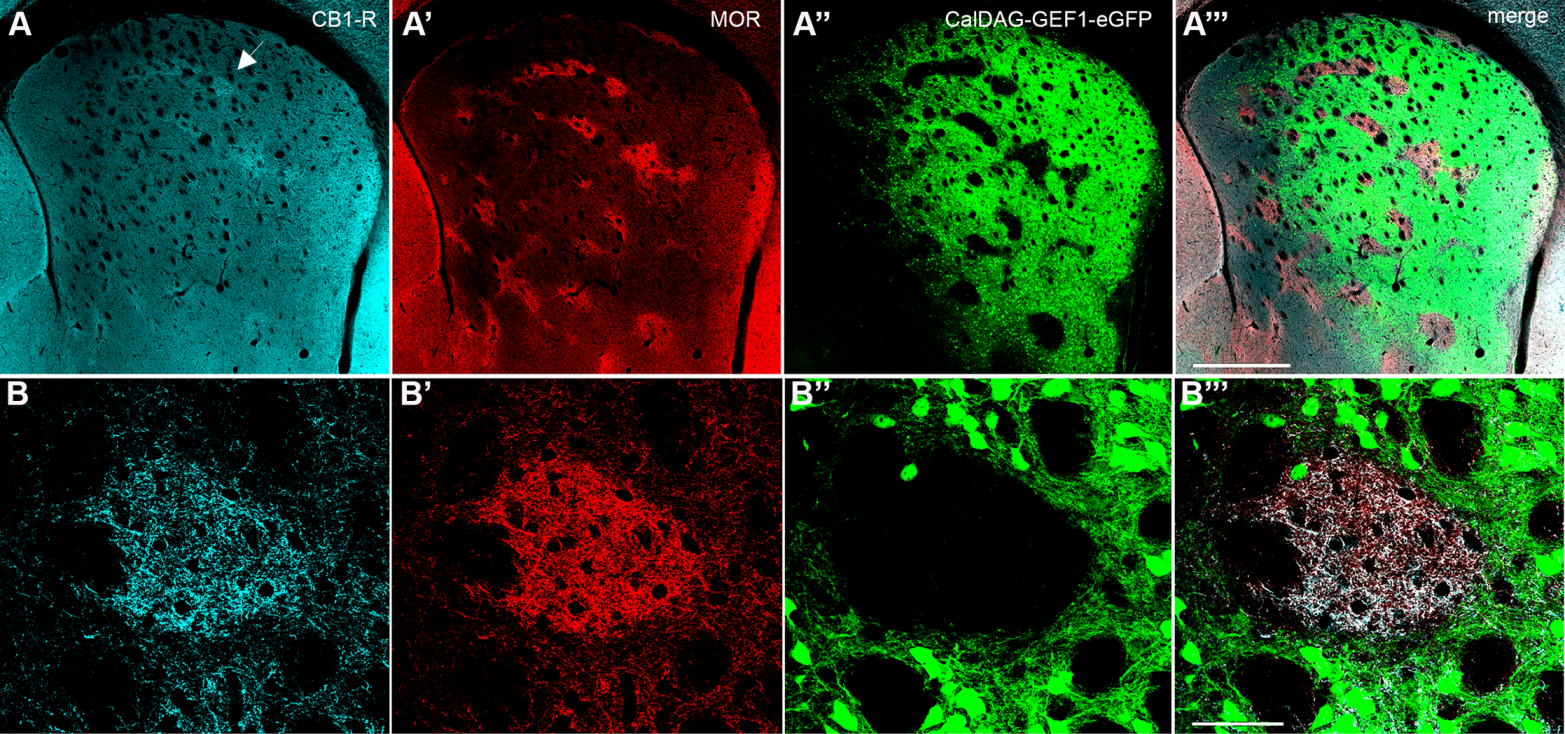
CB_1_-R immunoreactivity is enriched in striatal striosomes. A coronal hemisection through the left striatum shows immunofluorescence for CB_1_-R (A, B), striosomal MOR (A’, B’) and direct eGFP fluorescence in the matrix (A”, B”) in a CalDAG-GEFI-eGFP mouse, imaged by confocal microscopy. Merged images (A”‘, B”‘). The arrow in (A) designates the striosome shown in a magnified view (B-B”‘). Images were taken with the FV 1200 confocal microscope using the MIT immunohistochemistry protocol. Scale bar is 500 μm in A”‘, 50 μm in B”‘.

In serial sections co-labeled for CB_1_-R and MOR, CB_1_-R immunoreactivity was much weaker than MOR in the rostromedial striatum and medial striosomes (Fig 3A). In rostrolateral striosomes (near the associative-sensorimotor striatum border), MOR expression was greater than CB_1_-R expression, but the two showed some overlapping expression (Fig 3B). MOR expression was also high along the dorsal edge of the posterior limb of the anterior commissure in the mid-striatum, where CB_1_-R expression was low (Fig 3C). In the lateral striosomes of the caudal striatum, CB_1_-R was the dominant signal (Fig 3D). Similarly, triple immunofluorescence in CalDAG-GEFI-eGFP (matrix) mice for CB_1_-R and MOR, showed that CB_1_-R was enriched in central and rostrolateral striosomes at the associative/sensorimotor striatum border, relative to CB_1_-R in the surrounding matrix (Fig 4A and 4B).

**Fig 5:**
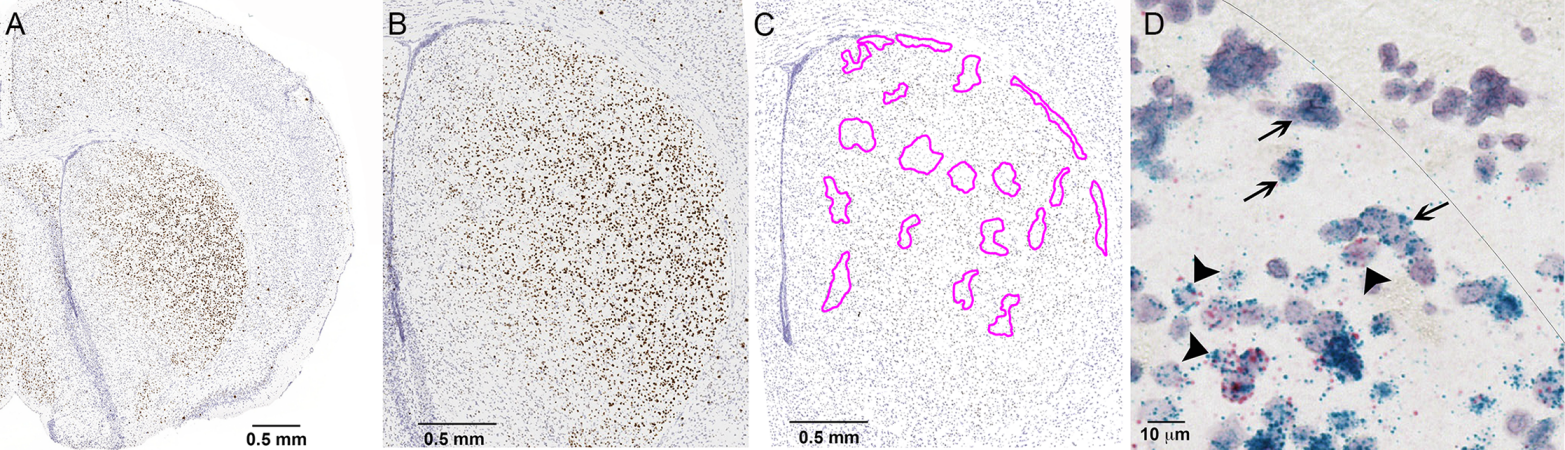
The cnr1 transcript is enriched in the dorsolateral striatum and is expressed in both striosomes and matrix. (A) *In situ* hybridization with a probe for cnr1 in a CalDAG-GEFI-eGFP mouse (with matrix-enriched eGFP) shows striatal enrichment of cnr1 in this coronal hemisection. (B) Magnification of (A). (C) In a section nearby to (A) in this matrix-eGFP mouse, *in situ* hybridization with anti-sense gfp probes was used to define striosomes (outlined in magenta), based on low probe density. Comparison of the striosome and matrix regions defined in (C) to the widespread distribution of cnr1 label in (B) indicates that cnr1 is expressed in both striosomes and matrix. Note the subcallosal streak, a striosome-rich region that lies just under the corpus callosum. (D) In a section that was labeled for both cnr1 (blue) and gfp (red), single-labeled cnr1-positive cells (examples at arrows) are visible within the striosome-enriched subcallosal streak, along the border with the corpus callosum (designated by a thin line), and double-labeled cells (examples at arrowheads) are visible in the matrix.

CB_1_-R immunoreactivity in axons in the striatal neuropil was diffuse, making it difficult to assess whether striosomal MSNs themselves were enriched in CB_1_-R. We therefore tested whether the *cnr1* transcript that encodes CB_1_-R was expressed in striosomes. CB_1_-R mRNA was grossly enriched in the dorsolateral striatum (Fig 5A), consistent with the protein distribution and receptor binding [7]. Higher-resolution imaging of striatal sections from a CalDAG-GEFI-eGFP mouse that were labeled for mRNAs encoding eGFP (matrix marker) and CB_1_-R demonstrated that CB_1_-R mRNA was abundant in both striosomal and matrix MSNs of the central and lateral striatum (Fig 5B–5D). Interestingly, others have noted a striosomal mRNA distribution that is distinctly heterogeneous in the medial striatum in rats [9]. Thus, MSN axons are a potential source of CB_1_-R immunoreactivity in the lateral striosomes, but protein gradients may be laterally shifted relative to the increased mRNA in the medial striosomes.

### Distribution of CB1-R in striosome-dendron bouquets of the substantia nigra

**Fig 6:**
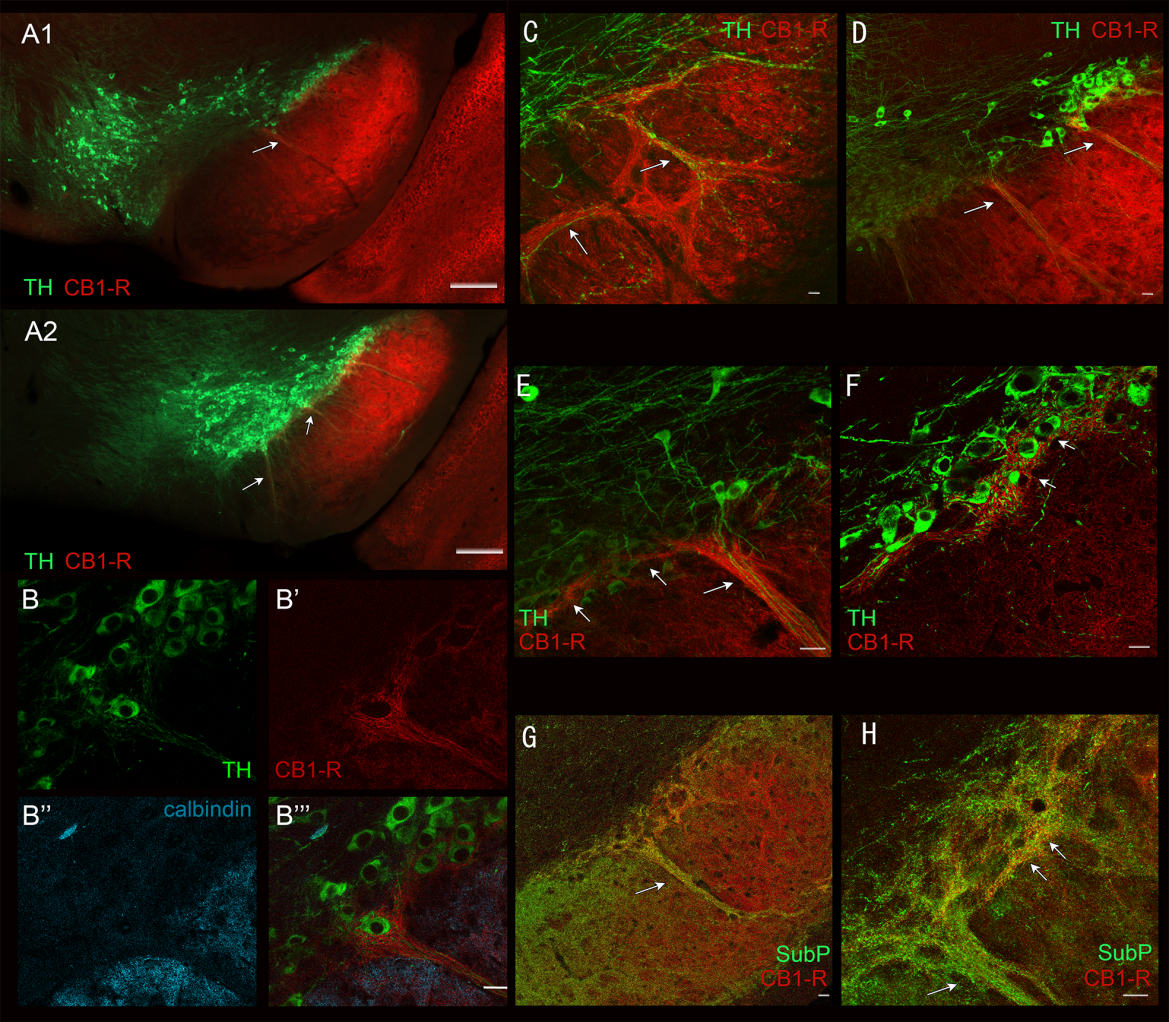
Lateral localization of CB_1_-R-dense striosome-dendron bouquets and ventral tier SNpc enveloping CB_1_-R-immunoreactive axons through the substantia nigra. Two low-power images (A1, A2) show apparent dendron structures and their location. Calbindin (blue) labels the fibers in the SNpr (B”) and avoids the SNpc and dendron (labelled by TH; B, B”‘). CB_1_-R labels the ventrally extending dendron (B’, B”‘). An example of a TH- and CB_1_-R-positive dendron in the rostral SN is shown in C. Low (D) and higher power images (E, F) of the lateral SN show a CB_1_-R-labelled dendron extend medially into the adjacent sector of SNc (arrows in E, F). A low-power (G) and confocal image (H) of CB_1_-R (red) and Substance P (green) shows overlap in the dendron bouquets. Scale bars are 20 μm in B-H. Low-power images were taken with the Axiozoom. Confocal images were taken with the LSM510.

Given these different distribution patterns, we next examined CB_1_-R expression in the SN, which receives partially segregated striosomal and matrix projections. Axons arising from striosomes strongly target groups of dopamine-containing neurons in the ventral tier of the SNpc, as well as their bundled ventrally extending dendrites, forming striosome-dendron bouquets [27]. In contrast, striatal axons from the matrix preferentially target the surrounding SNpr [27]. Bundles of ventrally extending dopamine neuron dendrites, as labeled by the dopamine cell marker tyrosine hydroxylase (TH), were spaced throughout the lateral SNpc (Fig 6A–6F). Images of sections through the SN at two levels relative to bregma are shown at low power (Fig 6A1 and 6A2), and in higher power (Fig 6B–6F), demonstrate the location, structure, and envelopment of ventral tier dopamine neurons in the lateral SN. Densely CB_1_-R-immunoreactive axons were detected in the ventral SNpc, often appearing to ramify hundreds of microns medial to the main dendron bouquet fiber bundles that climb along ventrally projecting dopamine neuron dendrites in these planes (Fig 6B, 6E and 6F, arrows), suggesting that the striosomal CB_1_-R-positive fibers ramify significantly into the ventral SNpc. Within the SN, the CB_1_-R immunoreactive bundles did not overlap with calbindin immunoreactivity (matrix axons, Fig 6B) but moderate colocalization was observed with Substance P in the lateral striosome-dendron bouquets (Fig 6G). The gradient of CB_1_-R immunoreactivity in the SNpr relative to Substance P was partially contrasting in the lateral-to-medial transition area of the SN, consistent with the CB_1_-R gradient observed in the striatum (Fig 6G and 6H).

**Fig 7:**
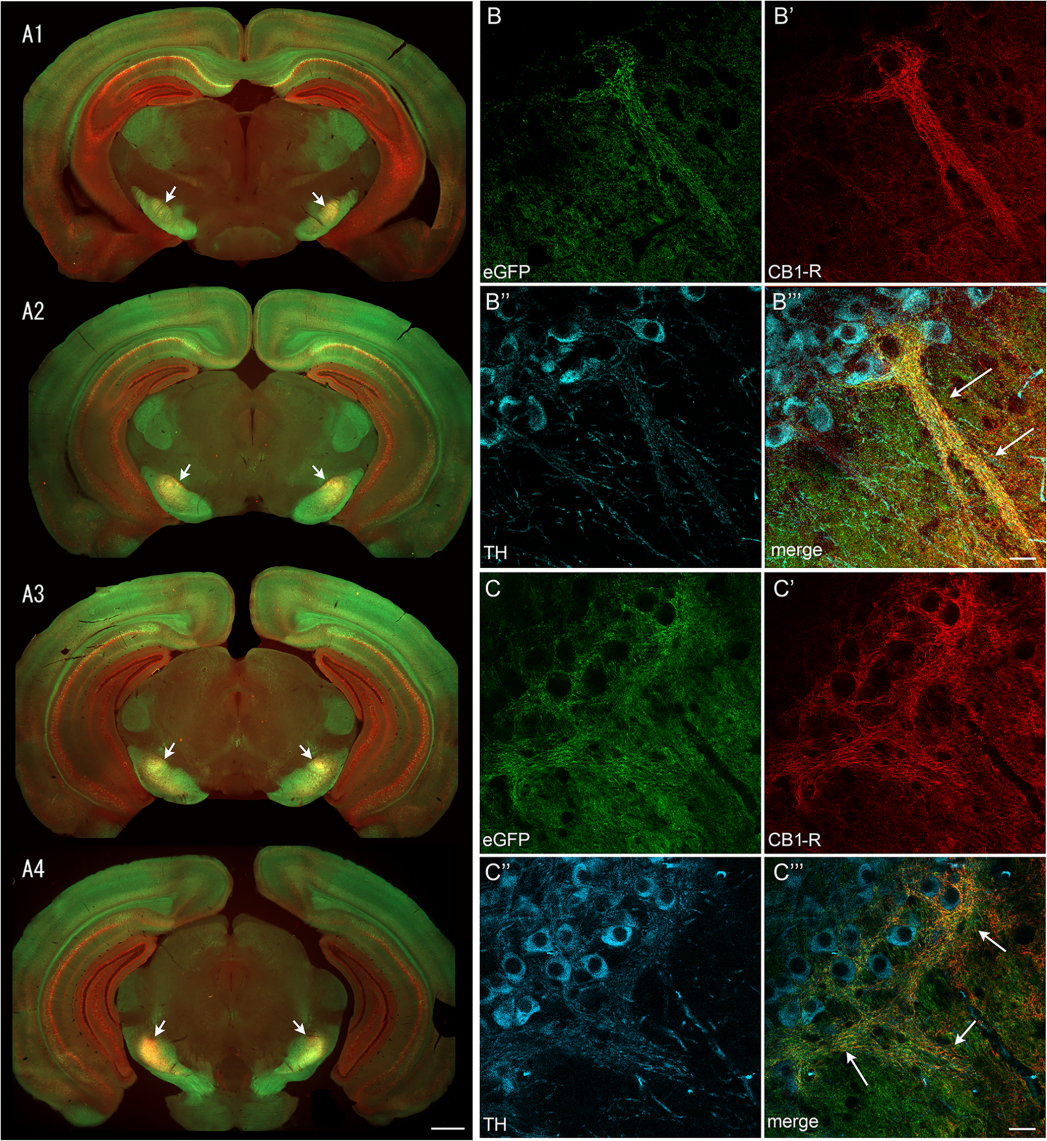
Dense CB_1_-R and Nr4a1-eGFP co-expressing striosome-dendron bouquets through the extent of the substantia nigra. The location and extent of Nr4a1-eGFP and CB_1_-R (red) overlap is shown in A1-A4 in serial sections. Two examples of Nr4a1-eGFP (B, C), and CB_1_-R (red, B’, C’) expressing axons enveloping TH+ neurons (blue, B”, C”) are shown at high power and merged in B”‘ and C”‘. Scale bars are 1 mm (A) and 20 μm (B, C). Low power images were taken with the Axiozoom microscope. B and C were taken with the LSM880.

When we used the Nr4a1-eGFP striosome-predominant signal to assess the extent of striatonigral projections with the CB_1_-R signal, regions of dense overlap were detected in discrete regions in the lateral SN (Fig 7A1–7A4, arrows). Imaging of the lateral SNpc co-labeled for TH highlighted 5–6 dendrons that contained Nr4a1-eGFP-, CB_1_-R- and TH-positive elements (example in Fig 7B and 7C for lateral and middle regions of SN, respectively). Imaging of the ventral SNpc revealed CB_1_-R positive fibers that were densely intertwined with the ventrally-extending TH-positive dendrites (Fig 7B and 7C). Furthermore, the presumed parent dopamine-containing neurons, just above the dendrons, as well as adjacent ventral tier dopamine neurons, were enveloped by CB_1_-R-positive axons (Fig 7B and 7C).

**Fig 8:**
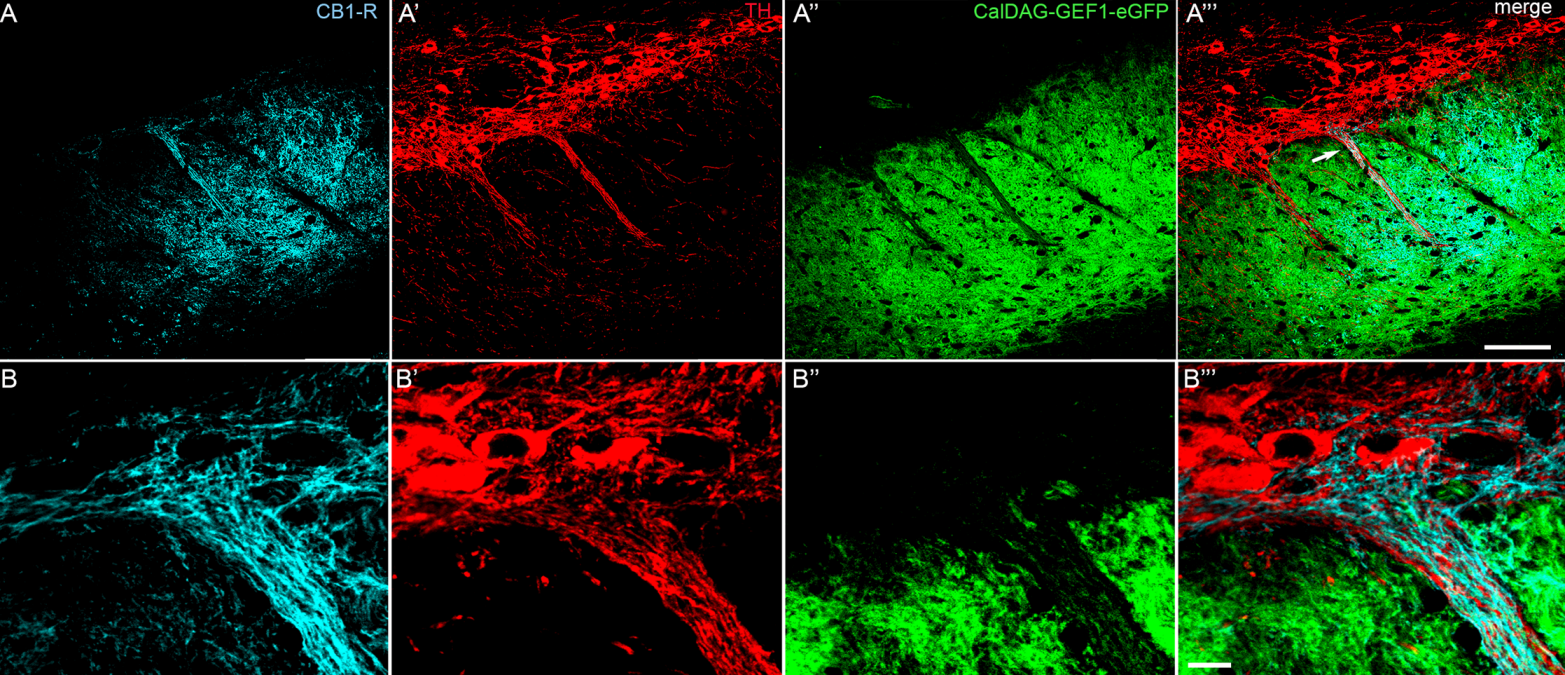
CB_1_-R immunoreactivity is enriched in striosome-dendron bouquets in the CalDAG-GEF1-eGFP matrix mouse line. A coronal hemisection through the left SN shows immunofluorescence for CB_1_-R (A, B), TH (A’, B’), and direct eGFP fluorescence in matrix MSN terminals (A”, B”) in a CalDAG-GEFI-eGFP mouse. Merged images (A”‘, B”‘) show CB_1_-R-positive fibers tightly entwined with dopamine-containing fibers that emerge from TH-positive cell bodies and extend ventrally into the SNpr, which is enriched for eGFP-positive axon terminals from matrix MSNs. Scale bar is 100 μm in A”‘ and 10 μm in B”‘. Images were taken with the FV1200 confocal microscope using the MIT immunohistochemistry protocol.

**Fig 9:**
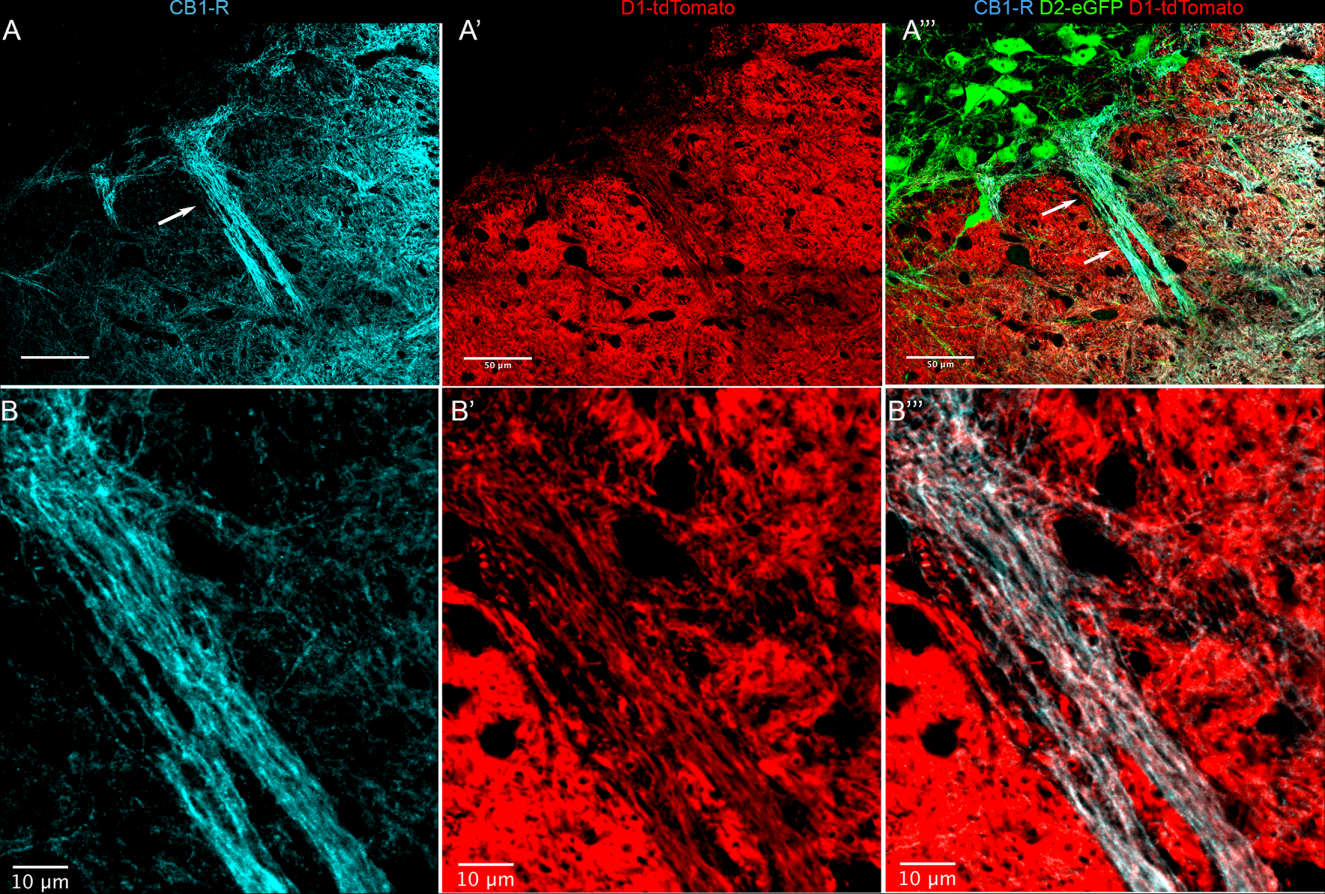
CB_1_-Rs are localized to Drd1-tdTomato fibers in the striosome-dendron bouquet. A coronal hemisection through the left SN shows immunofluorescence for CB_1_-R (A, B). Drd1-tdTomato in direct pathway MSN terminals (A’, B’), and a merge with Drd2-eGFP fluorescence that is in dopaminergic neurons (A”) in a double transgenic Drd1-tdTomato;Drd2-eGFP mouse. A single optical plane from a confocal image at higher magnification shows CB_1_-R and Drd1-tdTomato co-localization (white) in fibers within the striosome-dendron bouquet (B”‘). The arrow in (A) designates the dendron region shown in a magnified view (B-B”‘). Scale bar is 50 μm in A and 10 μm in B. Images were taken with the FV100 confocal microscope using the MIT immunohistochemistry protocol.

In contrast to the targeting of striosomal MSNs to SNpc observed in the striosome-enriched Nr4a1-eGFP mouse line, matrix MSNs tend to avoid the dendrons and show preferential innervation of the surrounding SNpr [24]. In SN sections from CalDAG-GEFI-eGFP (matrix) transgenic mice we observed preferential immunoreactivity for CB_1_-R in fibers that were within the eGFP-poor, TH-rich dendron bouquets (Fig 8). Complementary results were obtained when the Drd1-tdTomato:Drd2-eGFP line was stained for CB_1_-R (Fig 9). However, regions with heavy CB_1_-R immunoreactivity contained lower Drd1-tdTomato levels than the surrounding SNpr. This difference could be due to the large size of the bundled dopamine-containing dendrites within the bouquet structures. However, this was not the case with the Nr4a1-eGFP line (Fig 7), where eGFP is more highly expressed in Drd1 neurons [49], raising the possibility that the tdTomato signal reflects reduced Drd1 promotor activity in the dendron-projecting neurons in the lateral striatum.

These multiple lines of evidence based on well-characterized eGFP-expressing mice and multi-label immunofluorescence suggest that the increased CB_1_-R immunoreactivity in the striosome-dendrons is associated with MSN axons from the lateral striatum, where striosome-enrichment of CB_1_-R is prominent. The enrichment of CB_1_-R in the terminals within the SNpc is clear, but the density of the MSN axons, their heterogeneity, and expression of CB_1_-R on glutamatergic and interneuron terminals that might contribute to the immunoreactivity for CB_1_-R make it difficult to assign all CB_1_-Rs to a specific axon population in the striosomes.

### Dense striosomal CB1-R signal is on MSN collaterals

**Fig 10:**
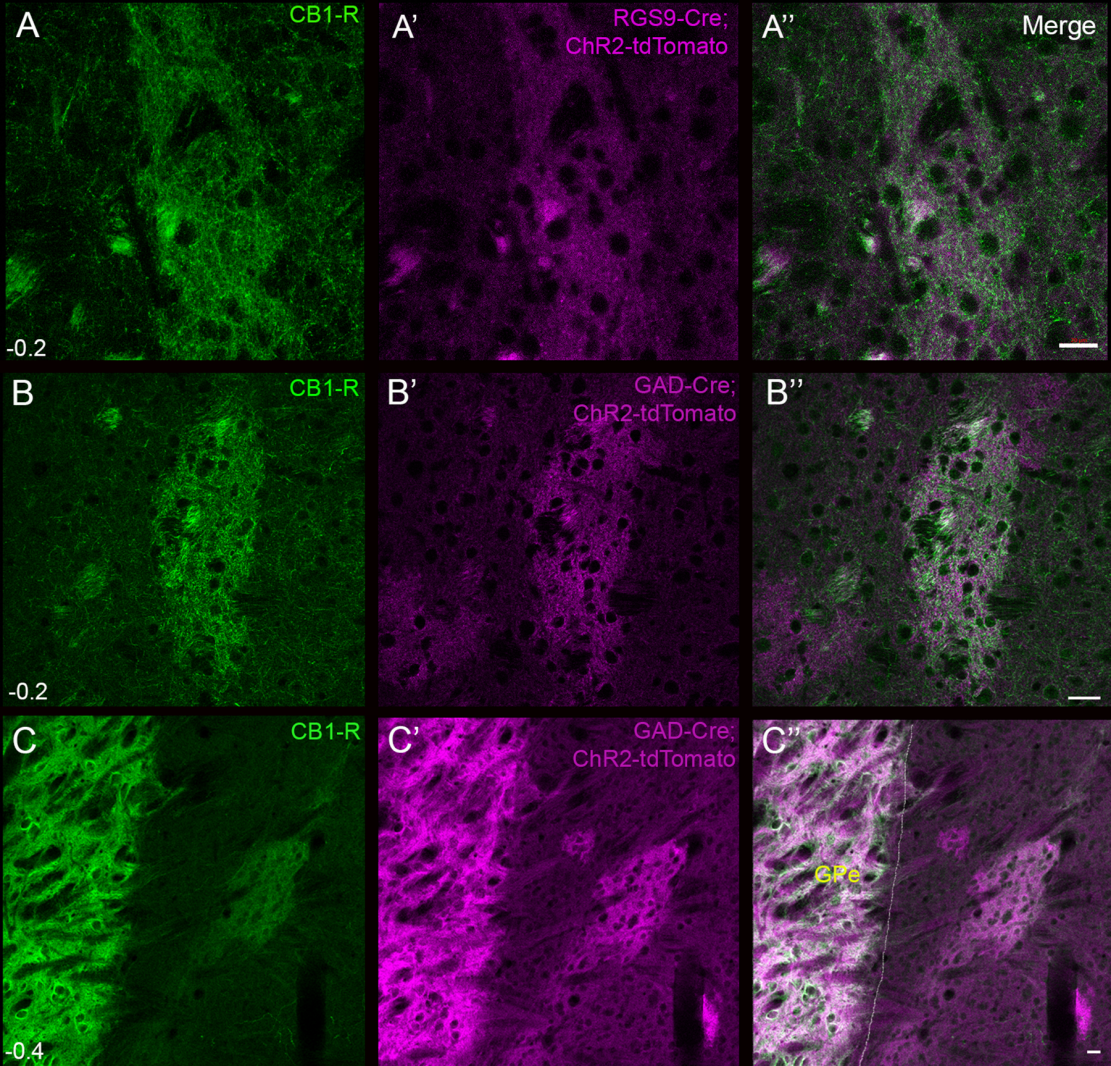
CB_1_-R labeling in the membrane of the RGS9-Cre:ChR2-tdTomato and GAD-Cre;ChR2-tdTomato reporters. CB_1_-R-immunoreactive MSN axon collaterals (A) and RGS9 promoter-driven tdTomato expression (A’) overlap (A”) in the dorsolateral striosomes. CB_1_-R-immunoreactivity and GAD promoter-driven tdTomato expression also overlaps in dorsolateral striosomes (B-B””) and in the globus pallidus (C-C”). Scale bars are 100 μm. Images were taken with the LSM510.

To confirm the source of CB_1_-R-positive axons in the striosomes, we turned to Cre-mediated membrane tdTomato expression (Ai27D), knockouts using cell-specific Cre transgenic mouse lines generated on the Nr4a1-eGFP background, and Cre-mediated reintroduction into a knockout strain. The Ai27D (channel rhodopsin, ChR2-tdTomato) reporter line [56] is useful for co-labeling of membrane proteins and fine axon collaterals (S5 Fig); therefore we stained two GABAergic/MSN Cre lines crossed to this reporter to examine co-localization with CB_1_-R in the diffuse MSN axons (Fig 10). The GAD-Cre mouse line also labels putative neurogliaform cells in the striatum and cortex (S2 Fig, arrows in 10B indicate neurogliaform cells). However, both GAD-Cre and MSN-selective RGS9-Cre-dependent ChR2-tdTomato expression filled the striosomes, striatopallidal fibers, and GP (Fig 10A–10C), which was strongly co-labeled for CB_1_-R. Because this reporter labels the entire cell, and the axons are small relative to the dendrites, the axonal colocalization appears as a haze in the background in these images.

**Fig 11:**
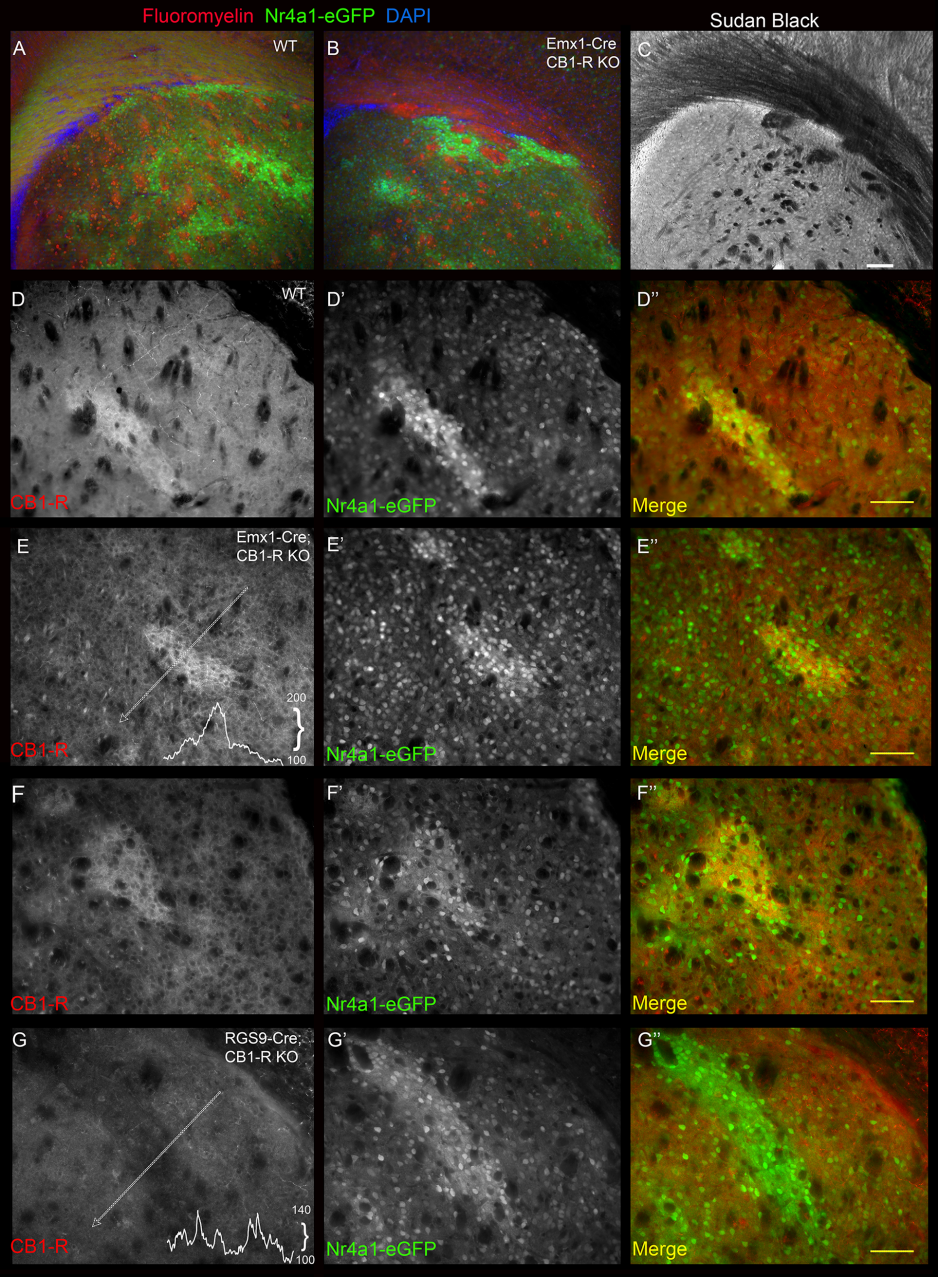
Emx1-Cre and RGS9-Cre-mediated CB_1_-R knockout on the Nr4a1-eGFP background reveal the source of CB_1_-R in striosomes. Fasciculation defects in the Emx1-Cre:CB_1_-R^f/f^ cross (A-C) illustrated with Fluoromyelin red (A, WT; B, Emx1-KO) and Sudan Black (C, Emx1-KO). CB_1_-R immunoreactivity was similar in striosomes in Emx1-Cre wildtype (D) and littermate Emx1-Cre;CB_1_-R^f/f^ knockouts (E). Nr4a1-eGFP (D’, E’) outlines the striosomes. Merged in D” and E”. RGS9-Cre wildtype (F) and littermate RGS9-Cre;CB_1_-R^f/f^ knockout (G) sections show a reduction in CB_1_-R immunoreactivity (G) in the striosome when CB_1_-Rs are deleted from striatal MSNs (G,’ Nr4a1-eGFP, merged in G”). Inset CB_1_-R density plots along the indicated line are shown for RGS9-Cre (C) and Emx1-Cre (E) knock out mice. These cohorts were examined between 25 and 30 days of age. Scale bar is 100 μm and applies to all panels. Wide field epifluorescence images were taken with the Axiovert microscope.

Next we employed cell-specific knockout strategies using neocortex-selective and MSN-selective Cre lines. CB_1_-R^f/f^:Nr4a1-eGFP mice were crossed with Emx1-Cre mice to determine if cortical neurons provide the source of CB_1_-R immunoreactivity we observed in the striosomes. Emx1-Cre mice express Cre in 91% of pyramidal neurons in cortex, sparse cells within striatum (S4 Fig) and [57], and therefore provide a useful tool for deleting CB_1_-Rs from glutamatergic neurons comprising the cortico-basal ganglia circuitry. When CB_1_-Rs were deleted from neocortical axons, mice displayed a fasciculation defect similar to the constitutive CB_1_-R knockout, yielding clumped fiber bundles in the striatum (Fig 11A–11C) [58, 59]. Nonetheless, dense striosomal CB_1_-R signal remained in the lateral striatum (Fig 11D). In sharp contrast, deletion of CB_1_-Rs from MSNs by crossing CB_1_-R^f/f^ mice with MSN-selective RGS9-Cre mice resulted in a reduction of striosomal CB_1_-R signal to near background levels (Fig 11G).

**Fig 12:**
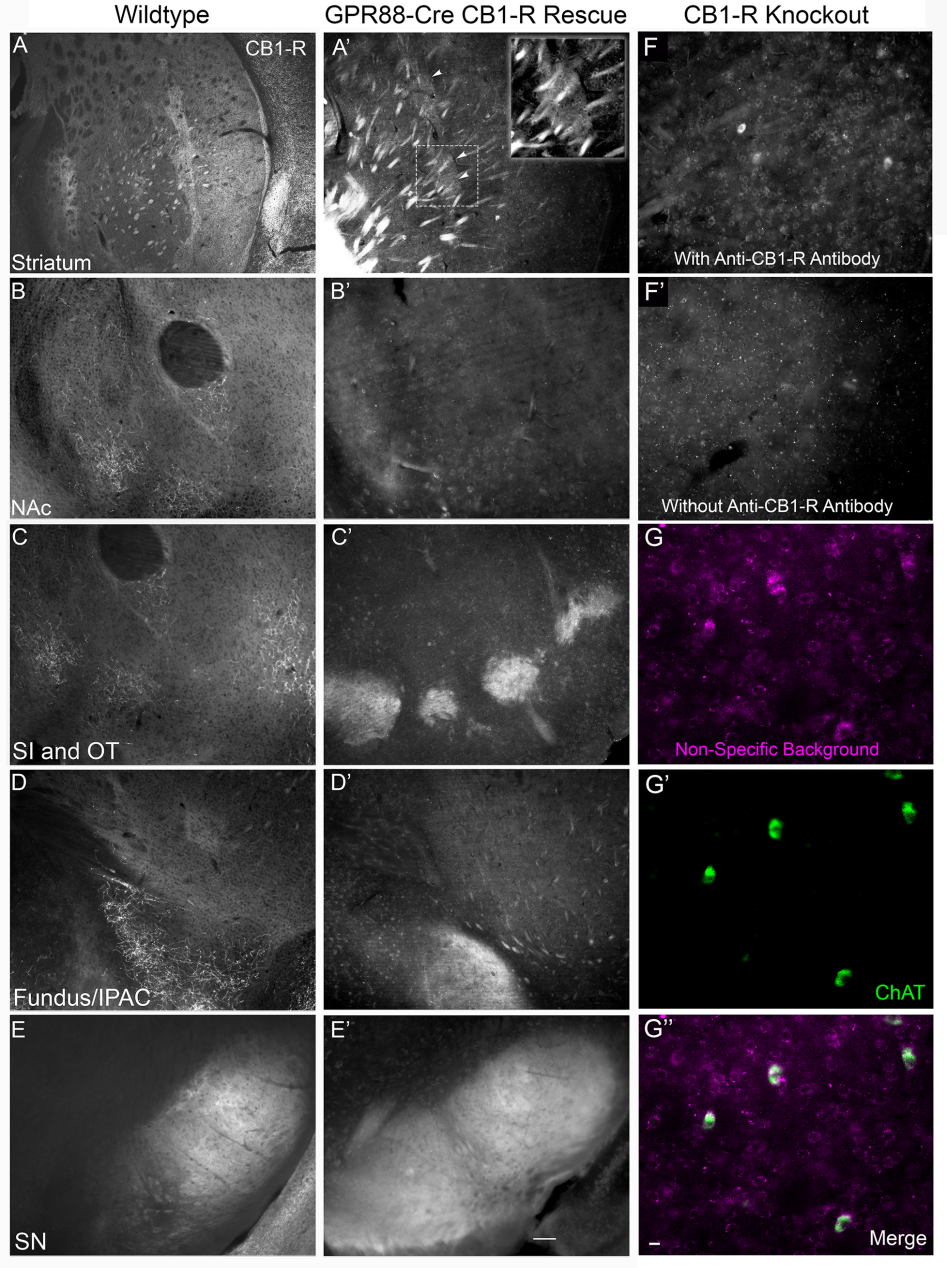
MSN-specific GPR88-Cre-mediated reintroduction of CB_1_-R recovers immunoreactivity for CB_1_-R in striosomes. Wildtype (A) is shown for comparison to the reintroduction in a littermate with GPR88-Cre-mediated CB_1_-R reintroduction (A’, inset shows higher power image of the boxed region). A faint signal was observed in the nucleus accumbens after reintroduction (B, B’). A strong signal was detected in the ventral pallidum/substantia innominata (SI; C’), and interstitial nucleus of the anterior commissure (IPAC; D’) that was not present in the wildtype (C, D). Reintroduction was robust but failed to recapitulate the normal gradient in the substantia nigra (SN; E, E’). Striatum at a similar caudal level to A and A’ in the CB_1_-R knockout (no GPR88-Cre-mediated reintroduction), with (F) and without (F’) the primary anti-CB_1_-R antibody. Background (non-specific) detection of cholinergic interneurons with the CB_1_-R antibody in the knockout (G-G”‘). Wide field epifluorescence images were taken with the Axiovert microscope. Scale bar in E’ is 100 μm and applies to A-F. Scale bar in G” is 20 μm.

As part of our normal characterization to confirm regional specificity of Cre expression in the RGS9-Cre mouse, these mice were crossed with Ai14 and Ai27D reporter lines, with the Cre allele carried by either the male or female parent. Regardless of the gender of the carrier, we observed Cre-dependent reporter expression in regions that had not been previously reported, including a particularly high level of expression in the medial prefrontal cortex (S5 and S6 Figs). The medial prefrontal cortex is known to project to striosomes, and contains CB_1_-R mRNA at low levels in projection neurons. Therefore, use of RGS9-Cre mice to assess the neuronal source of CB_1_-R in striosomes may be somewhat confounded by their extra-striatal Cre expression. Similarly, although the Emx1-Cre:CB1^f/f^ cross indicates that the source of the dense striosomal CB_1_-R signal is not likely cortical, CB_1_-Rs may be expressed in some striosome-targeting glutamatergic axons not represented in the Emx1-Cre-positive population of pyramidal neurons. Therefore, we sought to strengthen our evidence of an MSN source of striosomal CB_1_-R signal by assessing its expression pattern in transgenic global CB_1_-R knockout mice that have had CB_1_-Rs reintroduced into MSNs. Specifically, we crossed CB_1_-R knock out mice with a transgene insertion containing a lox-stop-lox sequence upstream of a CB_1_-R coding sequence with mice expressing Cre under the control of the MSN-enriched promoter, GPR88 [52], and performed immunohistochemistry on striatal brain slices from the offspring. This strategy allows for CB_1_-R expression only in cells that express GPR88 currently or at some point during development but does not rely on the endogenous CB_1_-R promoter. As predicted, GPR88-Cre-mediated rescue restored CB_1_-R immunoreactivity in striosomes and in both direct and indirect pathway projections (Fig 12A–12E’). Rescue failed to recapitulate the endogenous dorsolateral to ventromedial gradient. Because this genetic strategy does not differentiate between dorsal and ventral MSNs, we noted expression in projections to the ventral pallidum and a strong, hazy projection to the IPAC/fundus (compare to Fig 12D’ to Fig 12D). We assessed the background signal of CB_1_-R in the knockout by assessing the signal both with and without the primary CB_1_-R antibody (Fig 12F and 12F’). We also detected a non-specific somatic signal with the CB_1_-R goat and rabbit antibodies that labels cholinergic neurons in the striatum of the knockout mouse (Fig 12G). This signal was not particularly strong and was rarely observed in “wildtype” mice where the rare somatic signal was occluded by strong specific immunoreactivity in axons. CB_1_-R antibodies are notoriously non-specific [60], recognizing at least two other proteins, one being specific to mitochondria [61], and a second non-specific protein that has yet to be characterized.

### Cellular identity of MSN collaterals within the lateral striosomes and correlating MSN protein expression gradients

**Fig 13:**
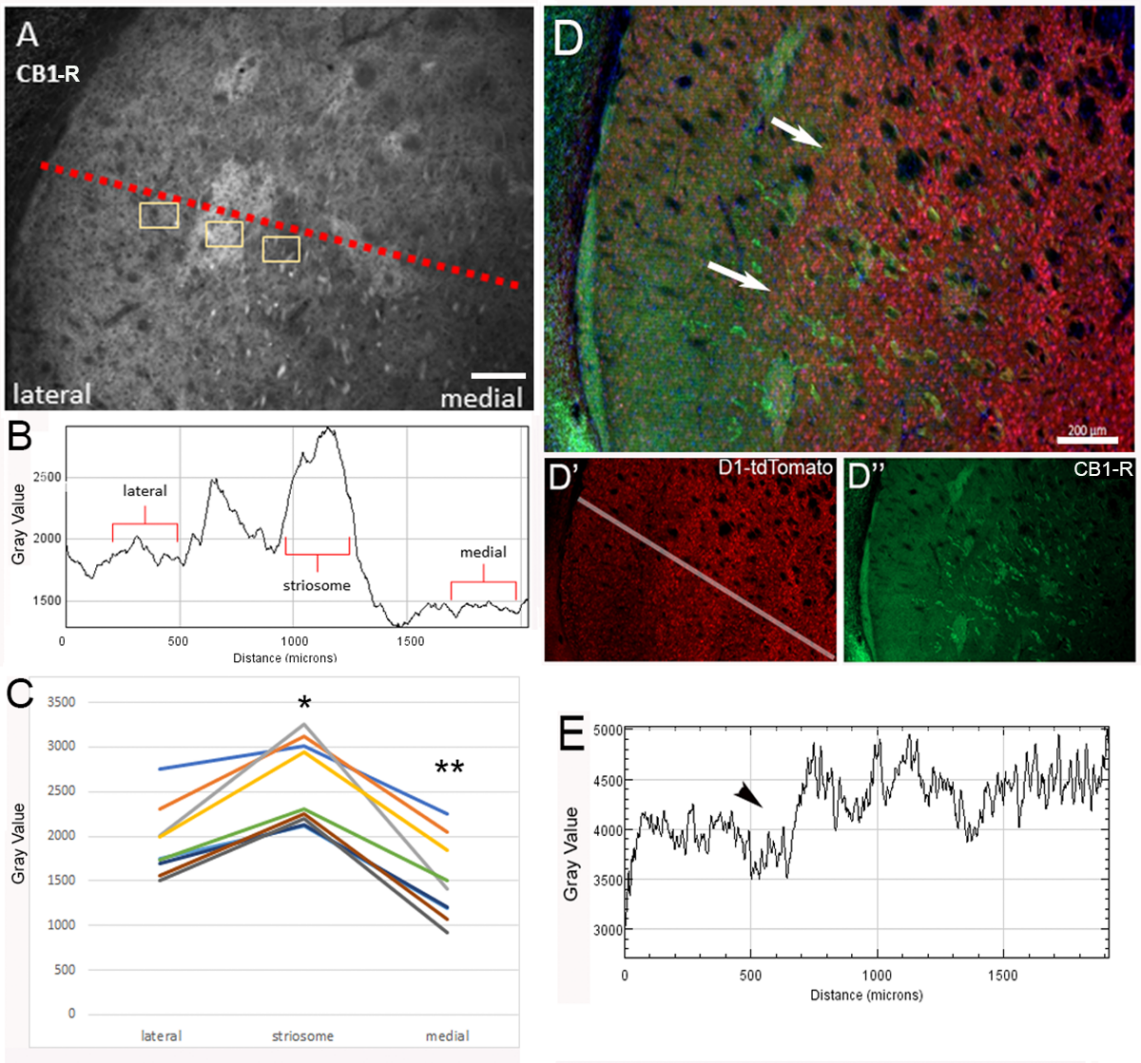
Gradient of CB_1_-R expression in the dorsolateral striatum. (A) A representative section immunostained for CB_1_-R with the angle and regions quantified. (B) A representative histogram of the mean intensity of a strip taken from this angle at a width of 500 pixels (1.028 pixels/ μm). (C) The optical density of the regions sampled along the dorsolateral-to-ventromedial gradient from nine individual slices from 6 mice sampled in the peri-commissural and post-commissural striatum are plotted as individual points. CB_1_-R expression gradient (D, D”) compared to D1-tdTomato expression gradient (D, D’). A representative histogram for D1-tdTomato levels is shown in E. Arrows in D and E indicate the change in the D1-tdTomato gradient. *p<0.001, different from lateral matrix; **p<0.001, different from striosome and lateral matrix. Images were taken at 10x with the Axiozoom microscope for quantification. Scale bar is 200 μm.

Multiple receptor and cell-type selective expression gradients are present in the striatum. Some groups have suggested that striosomes contain more Drd1-expressing cells, but this likely depends on the collection of striosomes examined, the marker used to define the striosomes, and the location within the striatum relative to bregma [55, 62, 63]. The average intensity of CB_1_-R expression was measured along a fiducial line in the caudal striatum (100 pixels in width, 1.028 μm/pixel, see S7 Fig for raw data) to quantify the dorsolateral-to-ventromedial gradient of CB_1_-R expression (Fig 13A, red dotted line). This method revealed a step-like elevation in expression at the striosome and a sharp decrease in expression in the adjacent medial matrix (Fig 13B). This sharp heterogeneity was particularly striking in the posterior striatum at the level of the GP (~bregma -0.8 mm). The average density of CB_1_-R signal in striosomes and adjacent lateral/medial matrix was quantified in nine different sections through the caudal striatum at the level of the GP in 6 mice (Fig 13A and 13C). The mean intensity of each region was compared, demonstrating less CB_1_-R expression in the matrix medial to the striosome that was significantly lower than the matrix lateral to the striosome, and consistent with a step-like pattern of expression in the medial matrix with a sharp transition at the striosome boundary. Interestingly, this CB_1_-R gradient was the inverse of a modest step-like gradient in Drd1-tdTomato expression in the caudal striatum (Fig 13D and 13D’; Drd1-tdTomato quantified with an intensity plot in E). Therefore, CB_1_-R expression is high where Drd1-tdTomato expression gradients begin to taper.

**Fig 14:**
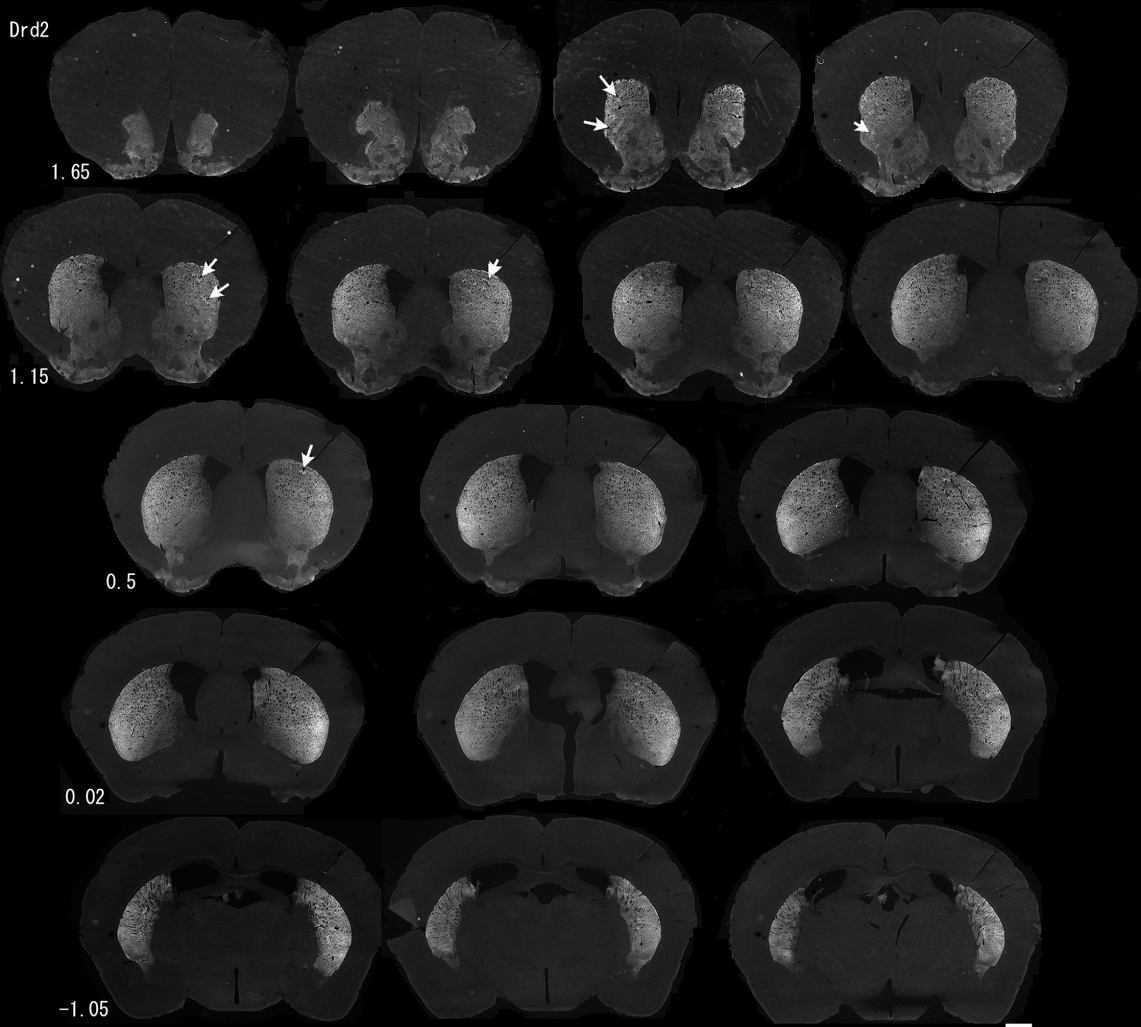
Gradient in Drd2 immunoreactivity through the striatum. Sections were taken at 160 μm intervals and numbers indicate the approximate location of the section relative to bregma. Arrows indicate examples of regions of increased immunoreactivity in rostral striosomes. Images were taken with the Axiozoom microscope.

The CB_1_-R gradient was similar to a modest gradient in Drd2-eGFP BAC transgene expression in the Drd1-tdTomato:Drd2-eGFP double transgenic mice (S3 Fig), where we also observed contrasting gradients of expression in fluorescent proteins. The CB_1_-R gradient was nearly identical to a dorsolateral-to-ventromedial gradient in Drd2 immunoreactivity in wildtype mice (Fig 14). This gradient is identical to classical Drd2 binding studies [64, 65] and immunohistochemistry using an antibody directed against the same epitopes in the third intracellular loop [66]. Faint striosomes with higher Drd2 immunoreactivity were visible in some sections (arrows, Fig 14) and in low-power images of double transgenic Drd1-tdTomato:Drd2-eGFP mice (S3 Fig), again consistent with the observations of Levey and colleagues [66]. It should be noted that the images presented in Fig 14 were taken under conditions that did not saturate the lateral signal; therefore, the Drd2 signal is low in the ventromedial striatum relative to the DLS, but this is not meant to suggest that the ventral striatum is devoid of Drd2 protein expression.

**Fig 15:**
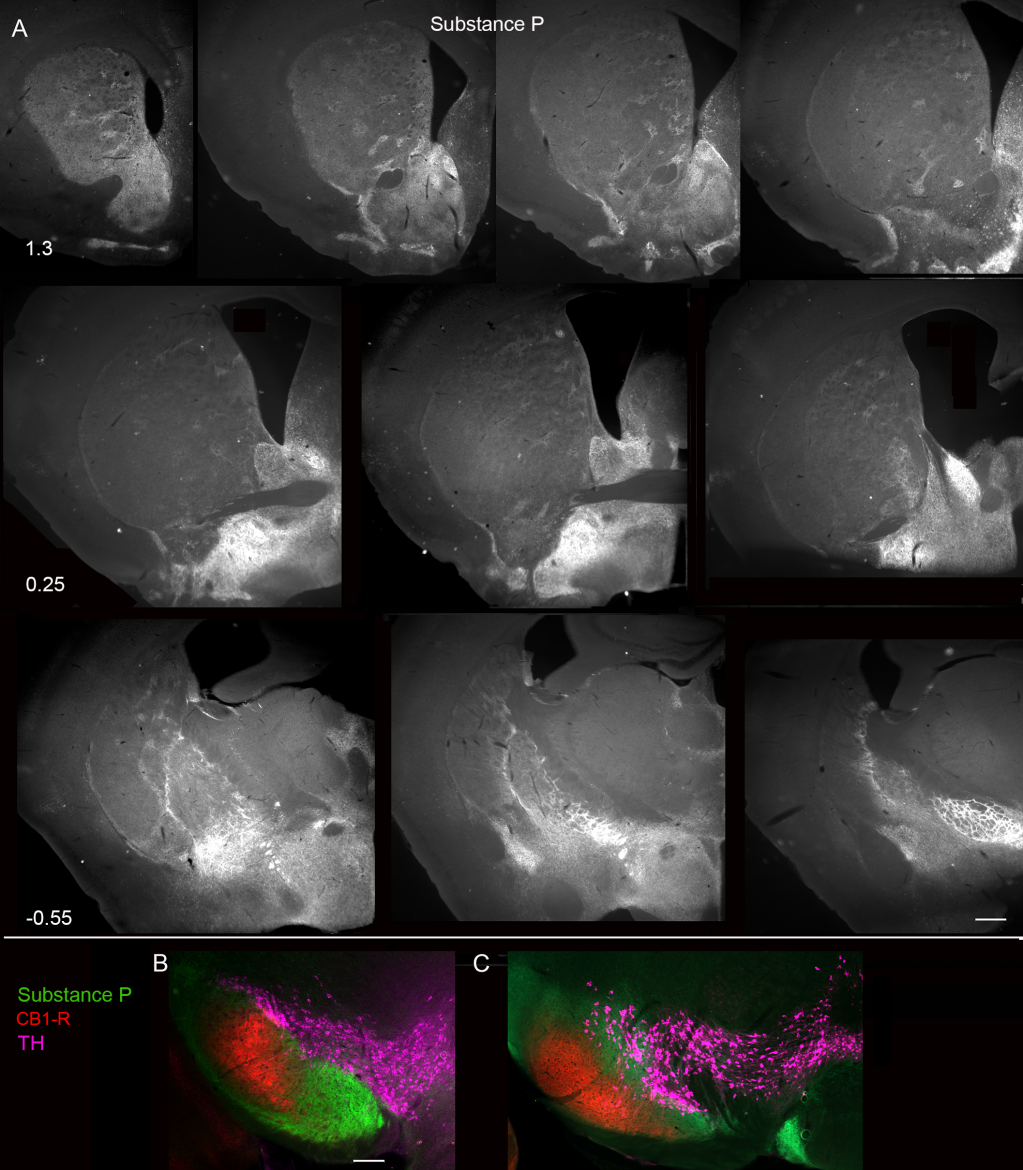
Ventromedial-to-dorsolateral gradient of Substance P immunoreactivity in the striatum. (A) Numbers indicate the approximate location of the section relative to bregma. (B, C) CB_1_-R, Substance P, and TH immunoreactivity in the SN at two levels. Scale bar in A is 500 μm. Scale bar in B is 200 μm and also applies to C. Images were taken with the Axiozoom microscope.

While the gradient in CB_1_-R expression was inverse to that of Drd1-tdTomato, a more pronounced contrast was observed in the gradients of CB_1_-R and Substance P immunoreactivity (Fig 15). Similar to other observations [55], Substance P immunoreactivity was detected in the rostral DLS, but the relative amount was lower than what was found in associative and limbic striatal regions. The marginal zone at the border of the striatum and the GPe, where Drd1-expressing cells form a distinct stripe, [63] and S3 Fig, was strongly immunoreactive for Substance P. This complementary CB_1_-R/Substance P expression pattern was maintained in the SN (Fig 15B and 15C). Therefore, CB_1_-R is likely expressed at higher levels by lateral Drd2-expressing MSNs, as the CB_1_-R gradient matches that of Drd2. The CB_1_-R distribution is largely inverse to that of the Substance P, and partially segregated from the Drd1-tdTomato signal by virtue of these complementary gradients. By extension, these data suggest that CB_1_-R expression is higher in axons that have lower (but detectable) levels of Substance P in the lateral SN (Fig 6G and 6H).

**Fig 16:**
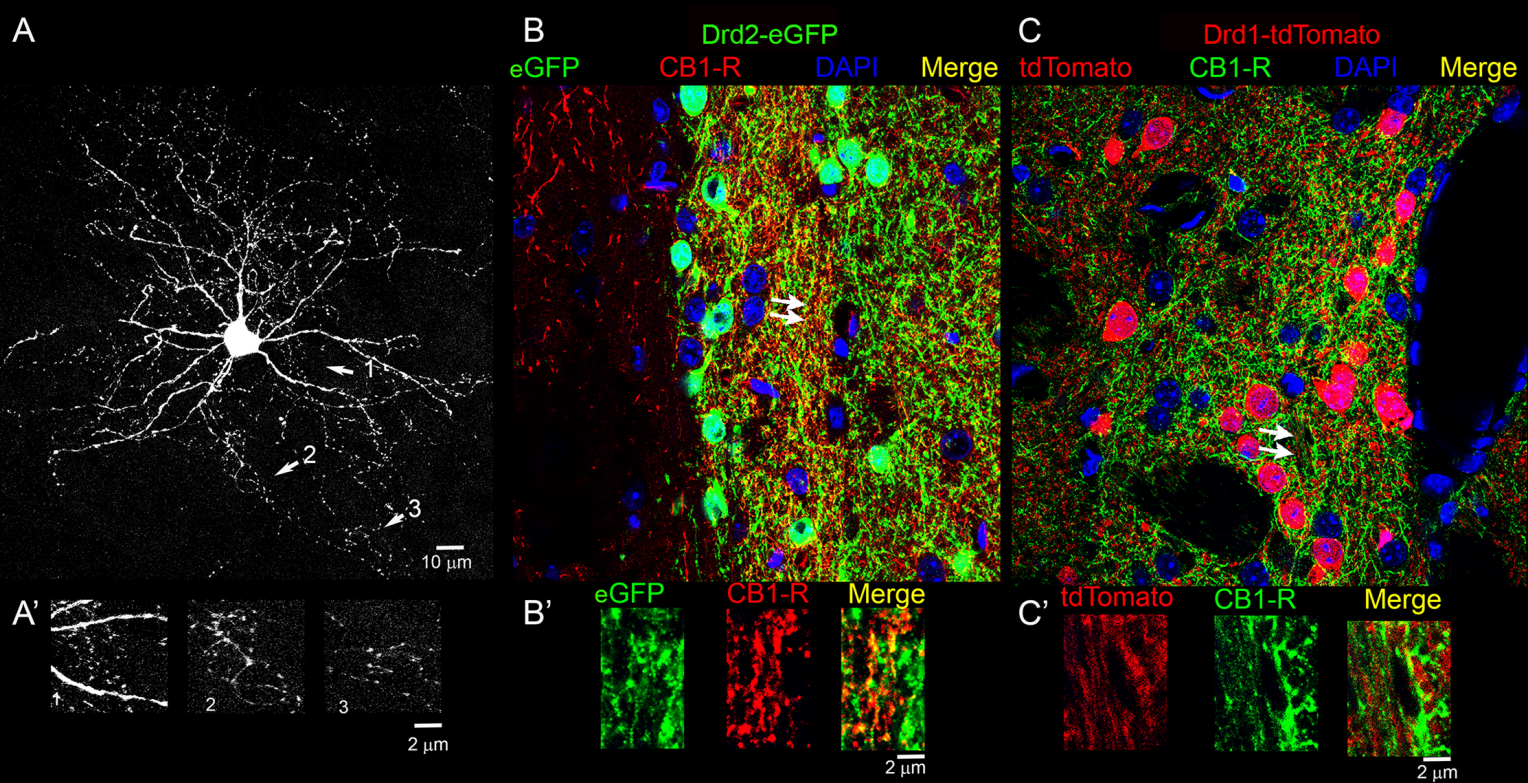
Projection images of CB_1_-R immunoreactivity in the Drd1-tdTomato and the Drd2-eGFP lines. (A) A representative tdTomato-filled MSN shown for axon scale. Areas indicated by arrows are shown magnified (A’) to demonstrate the size of the axons relative to the dendrites (scale bar in A’ is 2 μm). (B) CB_1_-R (red) and Drd2-eGFP (green) immunoreactivity in striatum. (C) CB_1_-R (green) and Drd1-tdTomato (red) immunoreactivity in striatum. Arrows indicate areas where single optical sections were expanded for B’ and C’. Yellow indicates colocalization and DAPI (blue) is used to define all nuclei in each strain. Scale bar in B is 10 μm and applies to C. Scale bar in B’, C’ is 2 μm. Images were taken with the LSM880.

Lastly, to further assess whether CB_1_-Rs are preferentially expressed in lateral Drd2-expressing MSNs, and whether this subclass of MSNs preferentially contributes to the high levels of CB_1_-R expression seen in striosomes, we again used transgenic mice expressing either tdTomato or eGFP under the respective control of Drd1 or Drd2 promoters. The C-terminal polyclonal antibodies against CB_1_-R detect at least two other proteins in addition to glycosylated and non-glycosylated CB_1_-Rs [60, 61], making initial attempts at high-resolution colocalization equivocal. Therefore, we turned to the recently available monoclonal antibody (Synaptic Systems) raised against the same C-terminal epitope. Fig 16A shows an example of a single dorsal striatal GAD-Cre-positive MSN infected with AAV9.CAG.FLEX.tdTomato, as described in [39], demonstrating the small size of the CB_1_-R-positive axon relative to the dendrites and somata, and the extensive local ramification of the axon. This example also highlights that axons are often incompletely penetrated by the fluorescent protein, adding another layer of difficulty to assigning CB_1_-Rs to a specific axon type. High-resolution confocal microscopy combined with double-label immunofluorescence in the caudolateral striatum showed CB_1_-Rs more closely associated with the Drd2-eGFP signal (Fig 16B) than the Drd1-tdTomato signal (Fig 16C). This bias was apparent in both the most lateral Drd2-enriched striosomes (Fig 16B) as well as more medial striosomes that are relatively more Drd1-tdTomato-enriched (Fig 16C). Single optical sections were examined at maximum resolution; in regions where clear axons were identified, Drd2-eGFP co-localized with CB_1_-R more often than with Drd1-tdTomato (Fig 16B’ and 16C’). In addition, Drd2-eGFP axons were apparent surrounding Drd1-tdTomato neurons and many of these axons contained CB_1_-R (Fig 16B). In contrast, Drd1-tdTomato and strongly CB_1_-R co-expressing processes surrounding tdTomato-negative cells were sparse in the caudal striosomes (Fig 16C), even in the more medial second “layer” striosome where Drd1-tdTomato expression is stronger.

## Discussion

The experiments presented reveal that CB_1_-Rs are expressed at high levels in striosomes in the dorsolateral and lateral striatum, where they are enriched in MSN collaterals. Further, CB_1_-Rs are highly expressed in the pallidal and nigral terminal fields of both direct- and indirect-pathway MSNs, and at particularly elevated levels in the striosome-dendron bouquets of the SN. In the SN, CB_1_-R immunoreactivity was pronounced in fibers within the ventral tier of the dopamine-containing SNpc, and in fibers bundled with the ventrally extending dopamine-containing dendrites of the bouquets. Given the proposed function of CB_1_-R in suppressing synaptic activity, the strongly graded striosome-predominant fields of CB_1_-R expression suggest that these receptors participate in “gating” and integrating output from the caudolateral striatum, which may be a function of the striosomal compartment.

### Gradients and striosome-matrix organization of CB1-Rs

The heterogeneity in CB_1_-R, Drd1-tdTomato, and Drd2 expression gradients illustrates the complexity of striatal anatomy in the mouse, which may significantly contribute to disparate observations between models and laboratories depending on the region examined, even within the same striatal sector. CB_1_-Rs in the striatum are striosome-enriched in a preferential lateral field of distribution that is more similar to Drd2 expression than Drd1, likely reflecting a bias towards greater striatal CB1-R expression by Drd2 MSNs. Multi-label immunofluorescence at high resolution supports such a configuration where Drd2-expressing MSN axon collaterals within the lateral striosomes contain higher levels of CB_1_-R than Drd1 MSN collaterals, however this will need to be confirmed with physiological experiments or high-resolution microscopy techniques. These dense CB_1_-R-expressing axons are also preferentially seen in the peri-somatic region around Drd2 MSNs in caudal regions of the lateral striatum, suggesting CB_1_-R may be more involved in homotypic inhibition in these striosomes. According to classical divisions, Drd1-expressing cells project to SN and Drd2-expressing cell to GP; therefore, the most parsimonious explanation is that the source of CB_1-_Rs in the lateral striosomes is the Drd2 population, while the Drd1-expressing neurons, possibly extending into medial striosomes [9], are the source of the SNpc projections.

This simplified interpretation is complicated by the observation that the direct and indirect pathways are not completely segregated and “bridging collaterals” arising from Drd1 cells also project to the GPe [67]. Recent work using the SepW1-Cre mice (striosome enriched Cre line), combined with retrograde rabies virus to map synaptic targets, indicates that striosomes do indeed project to the GP in an apparent topographical pattern where rabies injections into the DMS infects cells in the dorsal and medial part of the GPe [30]. Substance P immunoreactivity is strong in this region of GP (Fig 15), consistent with the gradient in the rostral striatum. It is therefore possible that the same cells that give rise to the dendron bouquets also give rise to the bridging collaterals. A small population of biphenotypic cells (Drd1 and Drd2 expressing) has been described in the developing striatum [68], but the paucity of these cells makes it unlikely that they contribute to the majority of the signal we observe in striosomes. Furthermore, we did not observe axons containing Drd1-tdTomato and Drd2-eGFP in the SN (Fig 9).

The accentuated patterns of CB_1_-R expression in caudolateral regions of the striatum are striking, as these are regions in which projections from motor cortex terminate in the extra-striosomal matrix [29, 39], suggesting that segregated functional modules exist in this region of striatum. The regional gradients of CB_1_-R were similar to those for Drd2 receptor expression, but more pronounced. The multiple gradients we describe, combined with the recent extensive characterization of gradients [55, 62, 63], confirm considerable heterogeneity within the striatum on which direct-indirect, lateral-medial, and striosome-matrix dichotomies are engrafted.

Our findings on CB_1_-R distribution in the striatum are suggestive of a disinhibition circuit, similar to that described for MOR [69–71], but with a different distribution pattern. GPCRs on presynaptic GABAergic terminals in striosomes inhibit GABA release, allowing postsynaptic cells to be more easily activated by glutamatergic afferents and/or modulated by dopamine. In the case of MOR, these regions may be enriched Drd1-expressing direct pathway neurons based on the expression gradients and previous MOR reintroduction experiments [70]. However, our data suggest that CB_1_-R rather than MOR may be the dominant presynaptic GPCR in the caudolateral striatum and may serve a predominant regulatory role in Drd2-expressing indirect pathway neurons in the lateral striosomes.

Reintroduction of CB_1_-Rs with GPR88-Cre in the stop-flox knockout mice implicates factors other than MSN identity in determining CB_1_-R expression, as has previously been suggested from unbiased profiling of single cells [72] and the gradient in CB_1_-R promoter-driven tdTomato expression [73]. After recombination, expression is no longer dependent upon the endogenous CB_1_-R promoter but instead expresses under the control of a strong promiscuous promoter. CB_1_-Rs were therefore present on all MSN axons in the rescue experiments. CB_1_-R is normally expressed selectively in striatopallidal fibers directed towards the GPe in a dorsolateral-to-ventromedial orientation, but not in fiber projections to the ventral pallidum, which are seen running dorsal-to-ventral with CB_1_-R immunoreactivity in the GPR88-Cre rescue experiments. Striato-ventral pallidal efferents are enriched in Substance P and their terminals have been traced back to associated limbic regions, the lateral habenula and VTA, which differentiate them from dorsal-ventral projecting striato-ventral pallidal fibers [74]. These ventrally projecting MSNs can be genetically “forced” to express CB_1_-Rs (as shown in Fig 12) and may express CB_1_-R transiently during development, but our findings, together with studies using CB_1_-R promoter-driven tdTomato [73], *in situ* hybridization [8–11], and reintroduction with GABA neuron-selective Cre lines [14] all suggest that this subset of MSNs do not express high levels of CB_1_-R under natural conditions. These experiments should also serve as a note of caution for reintroduction experiments where the introduced protein is no longer under control of the endogenous promoter.

### CB1-Rs and MSN communication within striosomes

CB_1_-Rs in striosomes are associated with a dense network of collaterals that surround MSNs, suggesting MSNs are their own targets. The gradient in Drd2 immunoreactivity and promoter-driven eGFP expression is consistent with CB_1_-R expression being greatest on terminals of Drd2-positive MSNs in the lateral striatum. In one study, functional coupling between recurrent collaterals in the dorsal striatum of young rodents indicate that collaterals are primarily from Drd2 MSNs synapsing onto Drd1 and Drd2 MSNs, and less common (only 6%) from Drd1 MSNs synapsing onto Drd2 MSNs [75]. Behavioral and pharmacological evidence exists for cooperation and cross-regulation of both Drd1 and Drd2 MSN function by CB_1_-R and some of these observations can be attributed to a presynaptic locus. Increased Drd2 expression occurs in CB_1_-R knockout mice [76], and CB_1_-R can also compensate for reduced Drd2 [77, 78], and regulate Drd2 MSN release of GABA in the GP [79, 80]. The existence of CB_1_-R and Drd2 heterodimers has also been postulated based on some of these observations [81]. *In vivo* THC exposure, however, suggests that CB_1_-R-mediated signaling is specific to Drd1-expressing cells in some regions of the striatum [82]. Given that the signaling to ERK is Drd1-dependent and that CB_1_-R is presynaptic, these data support a local striosome-related circuit whereby CB_1_-R activation allows for activation of Drd1-expressing cells by reducing local GABAergic inhibition of collaterals made by D2-MSNs, but the cells affected may differ depending on the region examined.

Other observations support a presynaptic locus and cooperation between MSN Drd2 and CB_1_-R in mediating the effects of psychomotor stimulants [83]. Similar activation patterns have also been seen with Drd2 antagonists [84], which can presynaptically regulate both GABA and dopamine release [79, 80, 85]. However, it has been reported that genetic deletion of CB_1_-Rs from MSNs blocks the effects of psychomotor stimulants on ERK phosphorylation, suggesting an enhanced GABA tone in these mice that is not compensated for with increased presynaptic MSN Drd2 activity. These *in vivo* pharmacological effects on ERK phosphorylation should be revisited in series and mapped onto distinct striosome populations, similar to experiments in multiple transgenic mouse lines addressing cellular and pharmacological heterogeneity in the NAc [86].

Given the differences in the distribution of CB_1_-R, Drd2 and MOR across striatal regions, it is possible that these presynaptic GPCRs serve similar but regionally tailored functions. Whether CB_1_-R and Drd2 or CB_1_-R and MOR at the motor/associative striatal border are acting together at the same synapses in the striosomes, or whether they regulate heterotypic transmission, remains uncertain. Multiple lines of evidence suggest that both CB_1_-R and Drd2 are capable of regulating MSN-to-MSN transmission [76, 87, 88]; both homotypic and heterotypic transmission are present between MSNs [75, 87, 88]. In the lateral striosomes, anatomical studies suggest a preferential enrichment of CB_1_-R in homotypic Drd2-to-Drd2 MSN collaterals; however, receptor expression does not always correlate with efficacy of G-protein coupling. Physiological studies will help resolve the components of these circuits, but they need to consider the striosome location and repertoire of complementary presynaptic GPCRs present in the striosome, as well as the varying density of MSN collaterals in the matrix and matrisomes.

### Possible functions of CB1-Rs in the striosome-dendron bouquets of the substantia nigra

From our findings, it seems highly likely that CB_1_-R receptors modulate function of the clusters of ventral-tier, dopamine-containing SNc neurons that give rise to the ventrally descending dendrons of the striosome-dendron bouquets. According to current understanding of the functions of endocannabinoids, their release from dopamine-containing neurons in response to activity could therefore inhibit, either acutely or via a long-term depression-like mechanism, GABA release from CB_1_-R receptor-expressing dendron fibers originating in MSNs of striosomes. Under physiological conditions, it is likely that any such endocannabinoid regulatory system between the striosomes, their local collaterals, and their nigral terminals would be carefully regulated by on-demand synthesis; however, under conditions of THC or cannabinomimetic drug exposure, the normal patterns of inhibition, disinhibition, and plasticity would likely be disrupted. This could change spike and oscillatory activity patterns within the striatonigral pathway. As a functional correlate, movement initiation requires appropriate coordinated firing within the basal ganglia that could easily be perturbed by excess cannabinoids [89]. A speculative but interesting working hypothesis raised by our findings is that CB_1_-R in the striosomal system in sensorimotor parts of the striatum and their projections to the ventral tier of SNpc may profoundly modulate motivated behavior under normal conditions and under conditions of excessive CB_1_-R stimulation. This also suggests that striosomes and their projections to the dopamine cells in the ventral SNpc may contribute to the impaired motivation and catalepsy that are characteristic of *in vivo* pharmacological cannabinoid activity.

Collectively, our data indicate that CB_1_-Rs are expressed at higher levels in striosomes, particularly in caudolateral regions of the striatum, and in the striosome-dendron bouquet projections to the SNpc. Cre-mediated reporter expression, loxP-mediated deletion, and Cre-dependent reintroduction indicate that MSN axons are the source of striosomal CB_1_-R immunoreactivity. Co-labeling experiments suggest that the Drd2 cells are the source of elevated striosomal CB_1_-R immunoreactivity in the caudolateral striatum, while Drd1 cells contribute to the elevated signal in the dendron bouquets. Further experiments will be required to elucidate the discrete and collective function of CB_1_-Rs within this heterogeneous basal ganglia circuitry, but these data refine our anatomical understanding of the striatal compartments, microcircuits, efferent targets, and principle cell types through which endogenous and exogenous cannabinoids can exert their effects on normal and abnormal basal ganglia physiology and behavior.

## Supporting information

S1 Fig Location of striosomes in the Nr4a1-eGFP mouse on the C57Bl6/J background. Sections are separated by 240 μm. Images were taken with the Axiozoom wide field epifluorescence microscope. Scale bar is 1 mm.
(TIF)

S2 Fig MSNs and axons from GAD_65_-ires-Cre expression pattern with a channel rhodopsin:tdTomato (Ai27D) reporter. Nuclei (DAPI) are shown in the blue channel. Striosomes (*) are larger than the nebulous neurogliaform cells present throughout the brain (indicated by arrows in some sections). Numbers indicate the approximate location of the section relative to bregma. Scale bar is 1 mm. Images were taken with the Axiozoom microscope.
(TIF)

S3 Fig Reciprocal gradients of Drd1-tdTomato and Drd2-eGFP expression in the striatum relative to bregma. Islands of distinct cellular segregation are present in the ventral structures near the accumbens (arrows). Striosomes indicated by “*”. Numbers indicate the approximate location of the section relative to bregma. Images were taken with the Lumar wide field epifluorescence microscope.
(TIF)

S4 Fig Emx1^Cre^ characterization using the zsGreen reporter. Cre expression was detected at low power (A, coronal through striatum, B, sagittal). Higher power images of cells in central amygdala (C) and striatum (D) are shown. Scale bars in top panels are 500 μm, 100 μm in C and 50 μm in D.
(TIF)

S5 Fig RGS9^Cre^ mediated recombination is detected in regions other than striatum when the signal from striatum is saturated. Imaging of the Ai14 tdTomato reporter under linear conditions (A) and conditions that saturate the striatum (B) reveals expression in adjacent brain regions. Compared to the soluble Ai14 reporter (C), expression in the ChR2 (Ai27D) tomato fusion is membrane associated (D) and does not fill the somata. Colabeling for calbindin (E,F, green) indicates that these regions of dense membrane tdTomato expression are striosomes. Scale bars in C-E are 100 μm, and E is 20 μm. Arrows indicate striosomes. Images were taken with the Lumar microscope (A,B) and the Axiovert (C-F).
(TIF)

S6 Fig Detection of RGS9^Cre^ mediated recombination throughout the brain. Sections were stained with an antibody against dsRed to amplify low level CAG-driven expression and imaged through the brain using the Axiovert and Lumar wide field epifluorescence microscopes. Approximate location is indicated relative to bregma. Abbreviations: **ac**, anterior commissure; **BLA**, basolateral amygdala; **BNST**, bed nucleus of the stria terminalis; **CeA**, central nucleus of the amygdala; **Cla**, claustrum; **CA1**, hippocampus cornu ammonis 1; **Den**, dorsal endopiriform; **DG**, dentate gyrus; **EP**, endopeduncular nucleus; **fr**, fasciculus retroflexus; **fx**, fornix; **GP**, globus pallidus; **Hb**, habenula; **ITC,** intercalated cells of the amygdala; **LGN**, lateral geniculate nucleus; **Lob**, lobule; **LPO**, lateral preoptic area; **LSr**, lateral septum rostral; **MOp**, primary motor cortex; **MRN**, median raphe nucleus; **MS**, medial septum; **MVN**, medial vestibular nucleus; **NAcC**, nucleus accumbens core; **NAcSh**, nucleus accumbens shell; **OB**, olfactory bulb; **Orb**, orbital cortex, **OT**, optic tract; **PAG**, periaqueductal grey; **PCG**, pontine central grey; **PF**, parafascicular thalamus; **PrL**, prelimbic cortex; **PT**, parataenial nucleus; **PVH**, paraventricular hypothalamus; **PVTh**, paraventricular thalamus; **SC**, superior colliculus; **sm**, stria medularis; **SN**, substantia nigra; **SSp**, primary somatosensory cortex; **St**r, striatum; **v3**, third ventricle; **v4**, fourth ventricle; **vl**, lateral ventricle; **VTA**, ventral tegmental area.
(TIF)

S7 Fig Raw data and statistical analysis used for Fig 13. (TIF)
