# Supplementary figures and images for: The cannabinoid-1 receptor is abundantly expressed in striatal striosomes and striosome-dendron bouquets of the substantia nigra
Source: PLoS One. 2018 Feb 21;13(2):e0191436. doi: 10.1371/journal.pone.0191436 (PMC5821318; doi:10.1371/journal.pone.0191436)

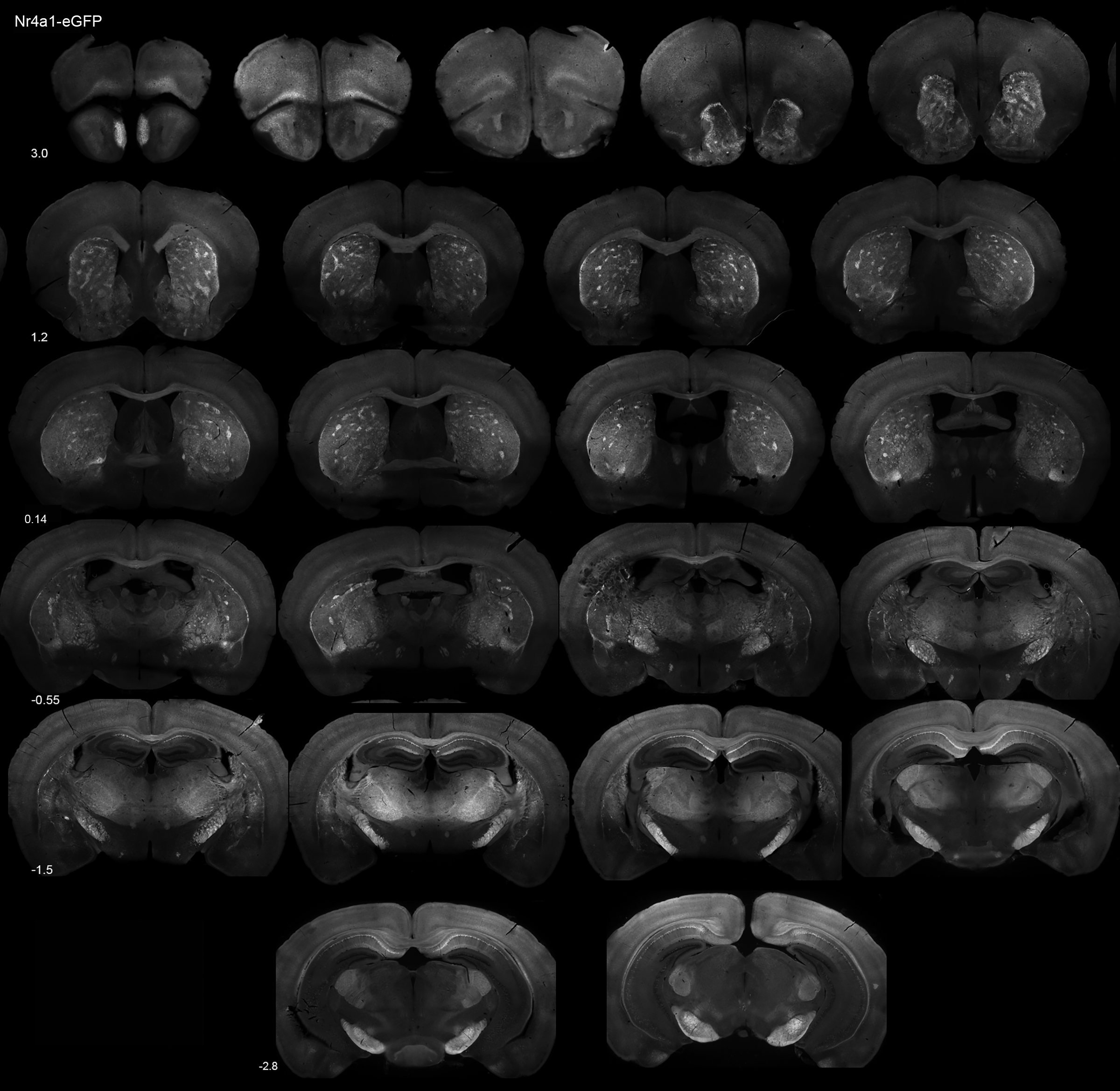

Supplement: S1 Fig — Sections are separated by 240 μm. Images were taken with the Axiozoom wide field epifluorescence microscope. Scale bar is 1 mm. (TIF) [file pone.0191436.s001.tif]

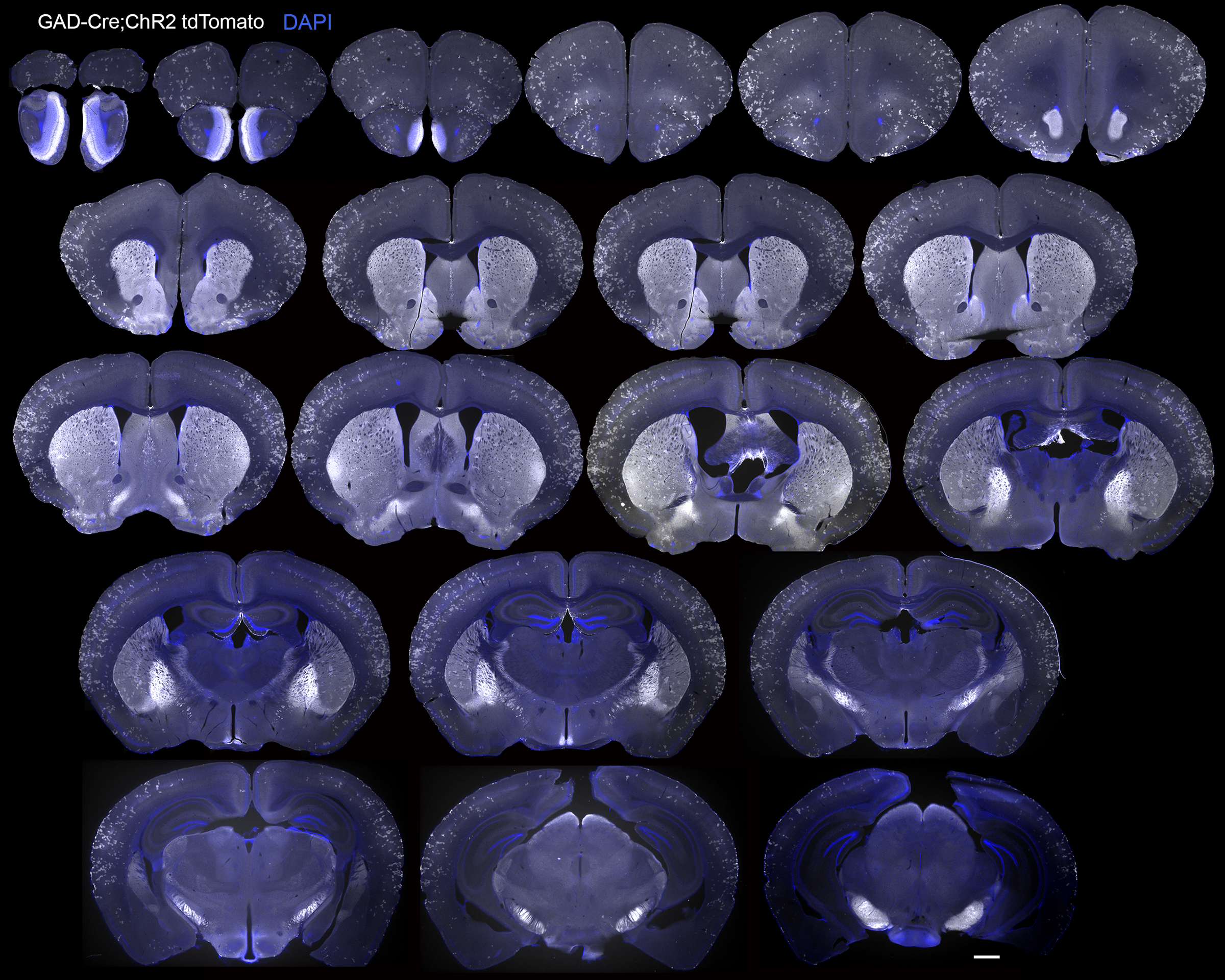

Supplement: S2 Fig — Nuclei (DAPI) are shown in the blue channel. Striosomes (*) are larger than the nebulous neurogliaform cells present throughout the brain (indicated by arrows in some sections). Numbers indicate the approximate location of the section relative to bregma. Scale bar is 1 mm. Images were taken with the Axiozoom microscope. (TIF) [file pone.0191436.s002.tif]

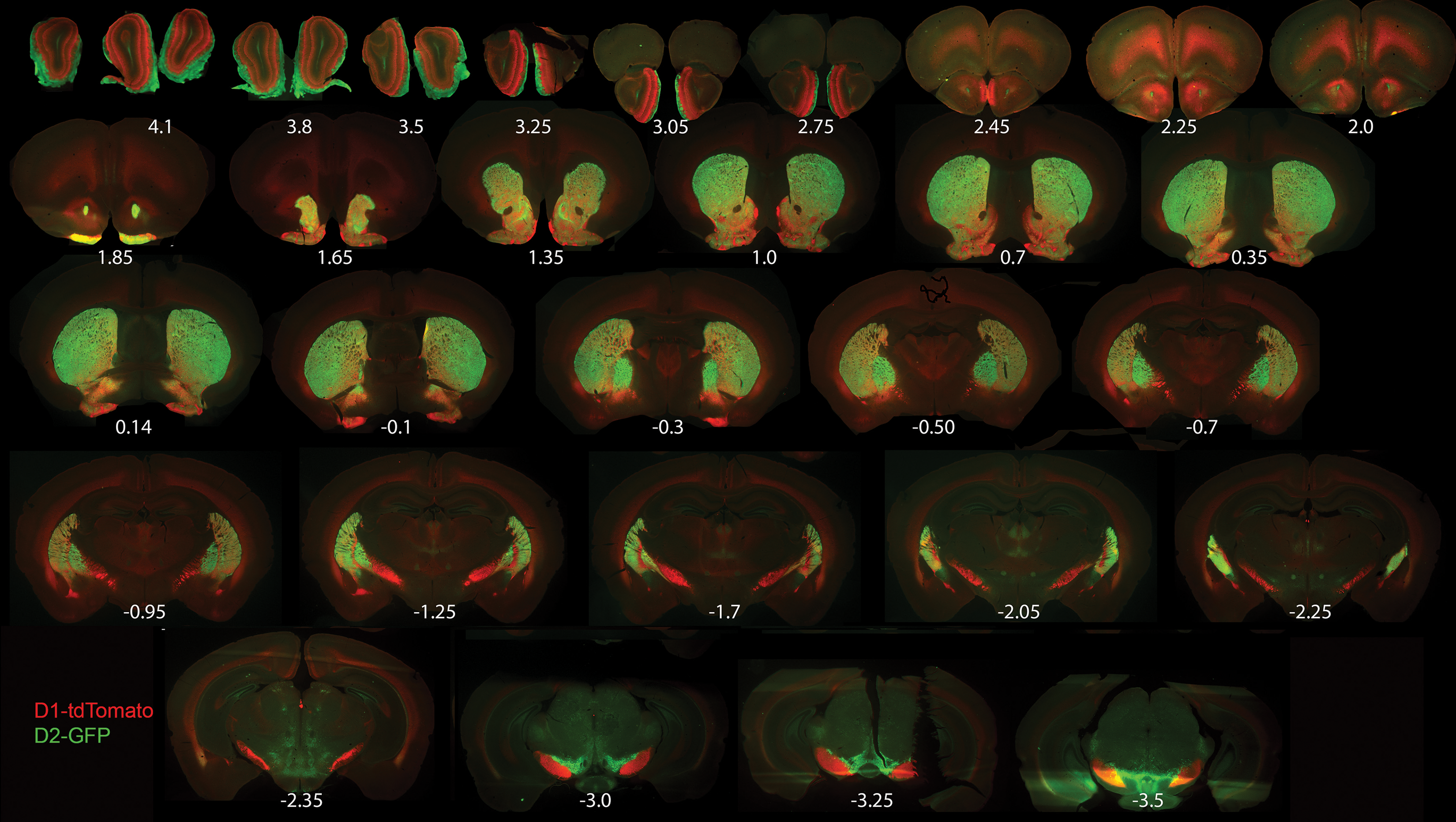

Supplement: S3 Fig — Islands of distinct cellular segregation are present in the ventral structures near the accumbens (arrows). Striosomes indicated by “*”. Numbers indicate the approximate location of the section relative to bregma. Images were taken with the Lumar wide field epifluorescence microscope. (TIF) [file pone.0191436.s003.tif]

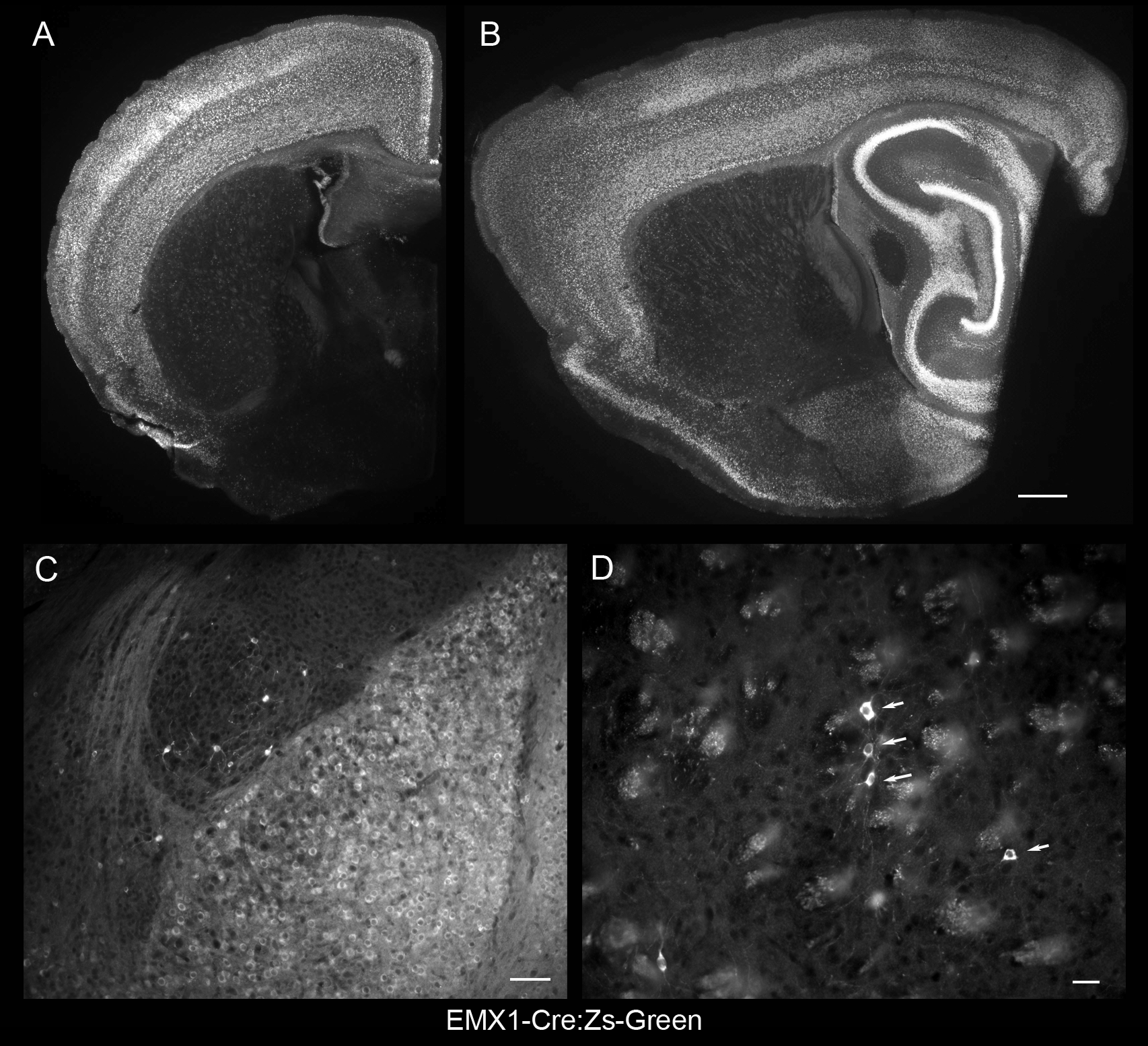

Supplement: S4 Fig — Cre expression was detected at low power (A, coronal through striatum, B, sagittal). Higher power images of cells in central amygdala (C) and striatum (D) are shown. Scale bars in top panels are 500 μm, 100 μm in C and 50 μm in D. (TIF) [file pone.0191436.s004.tif]

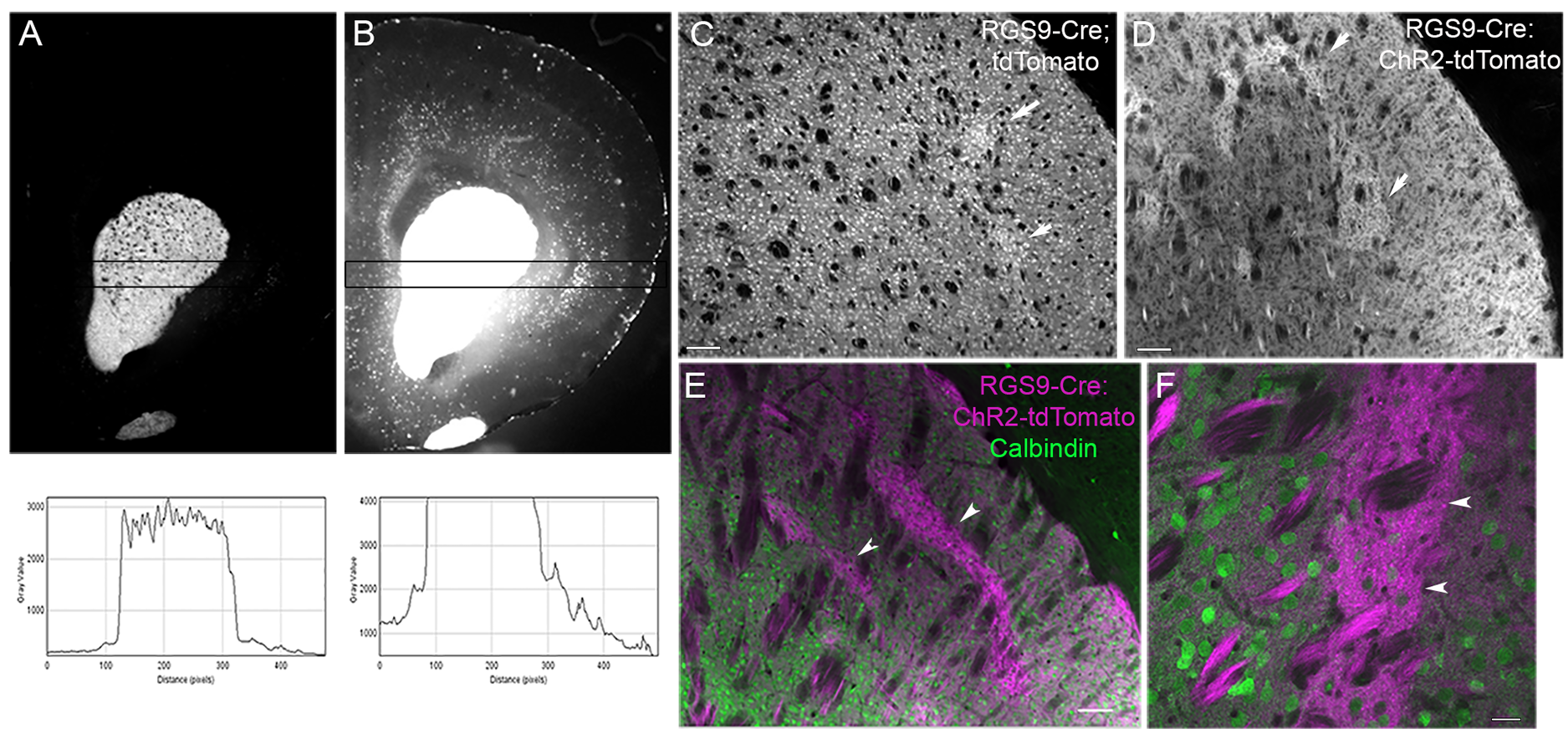

Supplement: S5 Fig — Imaging of the Ai14 tdTomato reporter under linear conditions (A) and conditions that saturate the striatum (B) reveals expression in adjacent brain regions. Compared to the soluble Ai14 reporter (C), expression in the ChR2 (Ai27D) tomato fusion is membrane associated (D) and does not fill the somata. Colabeling for calbindin (E,F, green) indicates that these regions of dense membrane tdTomato expression are striosomes. Scale bars in C-E are 100 μm, and E is 20 μm. Arrows indicate striosomes. Images were taken with the Lumar microscope (A,B) and the Axiovert (C-F). (TIF) [file pone.0191436.s005.tif]

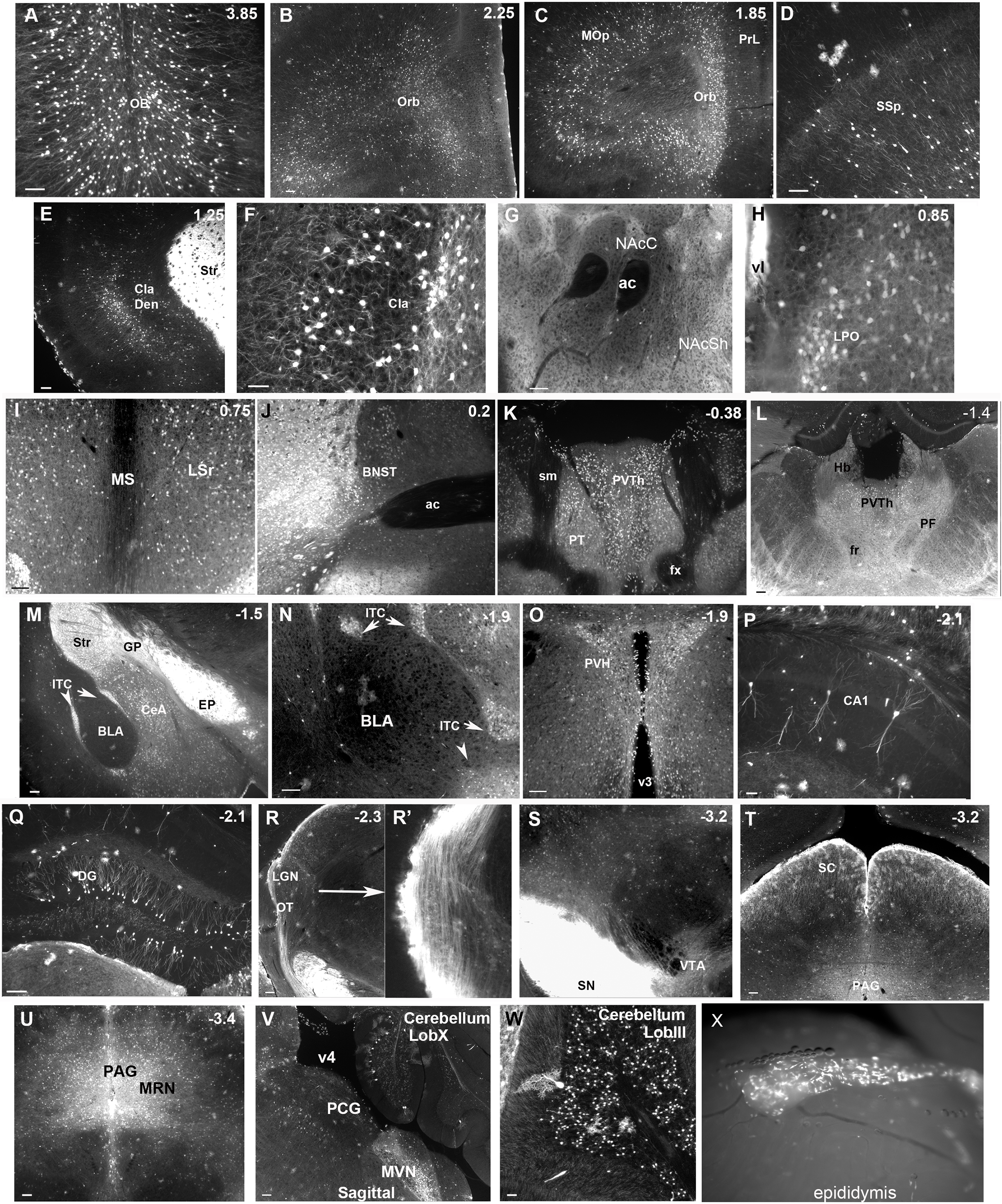

Supplement: S6 Fig — Sections were stained with an antibody against dsRed to amplify low level CAG-driven expression and imaged through the brain using the Axiovert and Lumar wide field epifluorescence microscopes. Approximate location is indicated relative to bregma. Abbreviations: ac, anterior commissure; BLA, basolateral amygdala; BNST, bed nucleus of the stria terminalis; CeA, central nucleus of the amygdala; Cla, claustrum; CA1, hippocampus cornu ammonis 1; Den, dorsal endopiriform; DG, dentate gyrus; EP, endopeduncular nucleus; fr, fasciculus retroflexus; fx, fornix; GP, globus pallidus; Hb, habenula; ITC, intercalated cells of the amygdala; LGN, lateral geniculate nucleus; Lob, lobule; LPO, lateral preoptic area; LSr, lateral septum rostral; MOp, primary motor cortex; MRN, median raphe nucleus; MS, medial septum; MVN, medial vestibular nucleus; NAcC, nucleus accumbens core; NAcSh, nucleus accumbens shell; OB, olfactory bulb; Orb, orbital cortex, OT, optic tract; PAG, periaqueductal grey; PCG, pontine central grey; PF, parafascicular thalamus; PrL, prelimbic cortex; PT, parataenial nucleus; PVH, paraventricular hypothalamus; PVTh, paraventricular thalamus; SC, superior colliculus; sm, stria medularis; SN, substantia nigra; SSp, primary somatosensory cortex; Str, striatum; v3, third ventricle; v4, fourth ventricle; vl, lateral ventricle; VTA, ventral tegmental area. (TIF) [file pone.0191436.s006.tif]

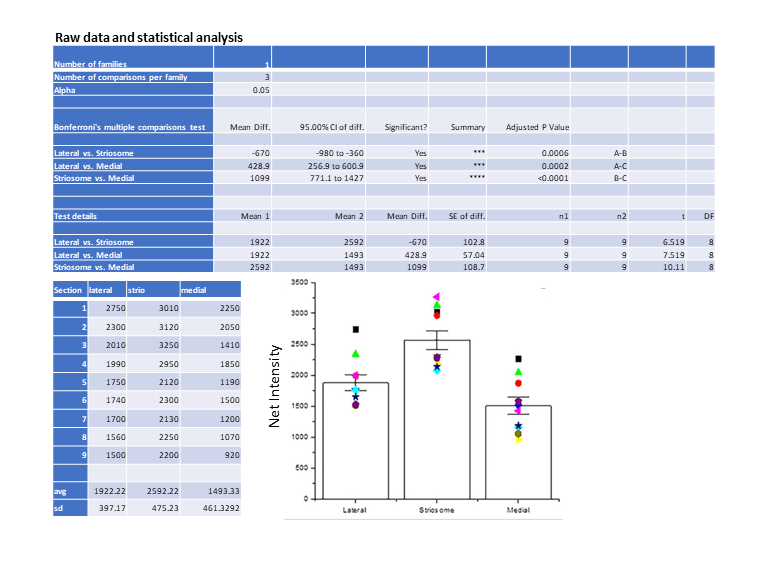

Supplement: S7 Fig — (TIF) [file pone.0191436.s007.tif]
